# Genotyping of *Brucella* species using clade specific SNPs

**DOI:** 10.1186/1471-2180-12-110

**Published:** 2012-06-19

**Authors:** Jeffrey T Foster, Lance B Price, Stephen M Beckstrom-Sternberg, Talima Pearson, William D Brown, Danika M Kiesling, Christina A Allen, Cindy M Liu, James Beckstrom-Sternberg, Frank F Roberto, Paul Keim

**Affiliations:** 1 Center for Microbial Genetics and Genomics, Northern Arizona University, Flagstaff, AZ, 86011-4073, USA; 2 Translational Genomics Research Institute, Flagstaff, AZ, 86001, USA; 3 Translational Genomics Research Institute, Phoenix, AZ, 85004, USA; 4 Idaho National Laboratory, Idaho Falls, ID, 83415, USA

## Abstract

**Background:**

Brucellosis is a worldwide disease of mammals caused by Alphaproteobacteria in the genus *Brucella*. The genus is genetically monomorphic, requiring extensive genotyping to differentiate isolates. We utilized two different genotyping strategies to characterize isolates. First, we developed a microarray-based assay based on 1000 single nucleotide polymorphisms (SNPs) that were identified from whole genome comparisons of two *B. abortus* isolates *,* one *B. melitensis*, and one *B. suis*. We then genotyped a diverse collection of 85 *Brucella* strains at these SNP loci and generated a phylogenetic tree of relationships. Second, we developed a selective primer-extension assay system using capillary electrophoresis that targeted 17 high value SNPs across 8 major branches of the phylogeny and determined their genotypes in a large collection ( *n* = 340) of diverse isolates.

**Results:**

Our 1000 SNP microarray readily distinguished *B. abortus*, *B. melitensis*, and *B. suis*, differentiating *B. melitensis* and *B. suis* into two clades each. *Brucella abortus* was divided into four major clades. Our capillary-based SNP genotyping confirmed all major branches from the microarray assay and assigned all samples to defined lineages. Isolates from these lineages and closely related isolates, among the most commonly encountered lineages worldwide, can now be quickly and easily identified and genetically characterized.

**Conclusions:**

We have identified clade-specific SNPs in *Brucella* that can be used for rapid assignment into major groups below the species level in the three main *Brucella* species. Our assays represent SNP genotyping approaches that can reliably determine the evolutionary relationships of bacterial isolates without the need for whole genome sequencing of all isolates.

## Background

*Brucella* are Gram-negative bacteria and the causative agent of brucellosis in domesticated animals, wildlife, and humans. Although the bacteria exhibit relatively strong host preference, separating the various *Brucella* species has proven extremely difficult due to minimal genetic differentiation [1]. Based on DNA-DNA hybridization, brucellae have previously been proposed to comprise a single species, with a series of biovars [2]. However, phylogenetic approaches explicitly incorporating host preference and virulence have upheld the six classical *Brucella* species: *B. abortus* (bovine), *B. melitensis* (caprine and ovine), *B. suis* (porcine), *B. canis* (canine), *B. neotomae* (desert woodrat), and *B. ovis* (ovine) [3-5]. Several new species have been recently described, including at least two species in marine mammals (*B. ceti* in dolphins, porpoises, and whales and *B. pinnipedialis* in seals) [6] and an additional species *B. microti* in the common vole ( *Microtus arvalis*) [7]. Other *Brucella* species undoubtedly exist within known and novel hosts [8-11].

The limited genetic differentiation and conservation within *Brucella* genomes has made genotyping a challenge. A promising approach that is capable of being incorporated into high-throughput assays is the use of single nucleotide polymorphisms (SNPs). Comparisons of *Brucella* genomes have revealed hundreds of SNPs that distinguish various strains [12-14]. Although the era of Next-Generation sequencing [reviewed in [15] is rapidly increasing available data for microbial genomic comparisons, full genome sequencing is currently not cost effective for genotyping large numbers of isolates and requires intensive bioinformatic efforts. Furthermore, in low diversity organisms such as *Brucella* only a small fraction of the nucleotides are polymorphic, suggesting that once rare polymorphisms are discovered, methods other than whole genome sequencing are more efficient for most purposes.

Molecular Inversion Probe (MIP) assays are an efficient and relatively inexpensive method of interrogating thousands of SNPs in large numbers of samples [16]. Although typically applied to research on human disease, the MIP assay can be readily applied to genotype SNPs in bacterial genomes. We compared four genomes from *B. abortusB. melitensis*, and *B. suis* to discover SNPs. We created a MIP assay to genotype 85 diverse samples and to discover canonical SNPs [17] that define *Brucella* species, strains, or isolates. We then created SNP-specific assays that use a Capillary electrophoresis Universal-tailed Mismatch Amplification mutation assay (CUMA) approach for major branch points in the phylogeny and screened them against a large and diverse collection of isolates ( *n* = 340). Finally, we compared these results to 28 *Brucella* whole genomes *in silico* to place our genotyping into context with all major biovars and isolates.

## Results

**Figure 1:**
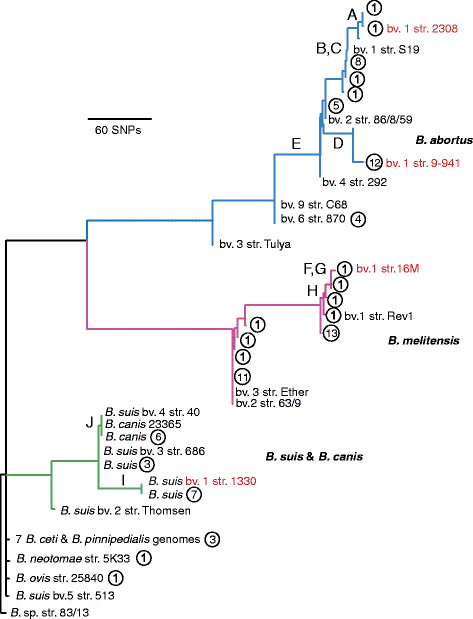
***Brucella*phylogeny based on comparison of 735 single nucleotide polymorphisms screened using Molecular Inversion Probes (MIP) in 85 samples and then compared to those SNPs in 28 whole genome sequences, which are the named isolates in the tree.** Discovery genomes are indicated in red. Letters on branches refer to phylogenetic locations of CUMA assays developed in this work and genotyped against DNA from a diverse collection of 340 isolates. Circled numbers indicate the number of isolates with identical MIP genotypes (allelic profiles) at that branch location.

A total of 833 MIP probes consistently amplified their target sites across 85 samples. Among these probes, 777 identified truly polymorphic sites. This dataset contained only 4% missing data (2,636 no calls in 66,045 SNPs), where no SNP was determined at a particular locus for a sample. Comparisons to SNPs from 28 whole genomes yielded 735 SNPs in our phylogenetic analysis (Figure 1), allowing for placement of these genomes within the MIP tree and *in silico* determination of their SNP alleles. The full MIP tree with all 777 loci and 85 samples, excluding the whole genomes in the comparisons, is also given (Additional file 1: Figure S1).

Our MIP assay distinguished *B. abortus*, *B. melitensis*, and *B. suis*; the three prominent *Brucella* species (Figure 1). A total of 524 SNP loci had complete allele calls (i.e. no missing data) across all 85 samples. The assay strongly differentiated *B. melitensis* and *B. suis* into two clades each. Within *B. melitensis*, at least 27 SNPs on branch H separate strain 16 M and its related isolates in biovar 1 from isolate 63–9 and related isolates of biovars 2. Subsequent analyses (see below) group biovar 3 with biovar 2 isolates. Based on these data, the assay for branch H appears to be specific to *B. melitensis* biovar 1. The two clades in *B. suis*, denoted by branches I and J, included all isolates of in the species except for biovar 5, which was distantly related to other members of this species. Some isolates from *B. suis* are more closely related to *B. canis* isolates (branch J) than to other *B. suis* isolates (branch I), indicating that *B. suis* is a paraphyletic species. Of the SNPs with complete genotyping data, at least 31 SNPs on branch I separate *B. suis* 1330 and related isolates from *B. canis* and related *B. canis* and *B. suis* isolates. However, no SNPs uniquely identified *B. canis*. *Brucella abortus* was even more differentiated, and can be divided into at least four distinct clades. Samples from *B. abortus* biovar 1, which contains the two SNP discovery strains, plus the type strain for biovar 2 (strain 86/8/59), make up the majority of samples and diversity within the *B. abortus* clade. All were found on branch E, which was further divided into branches A-D. Samples from the other *B. abortus* biovars are more distantly related and form distinct branches. As expected, the other species in the assay, including *B. neotomae*, *B. ceti*, *B. pinnipedialis*, and *B. ovis* were poorly distinguished from each other. Missing data for SNP loci caused the differences in branch lengths that are seen between Figure 1 and Figure S1.

CUMA assays verified the SNP alleles for all 85 of the samples run in the MIP assay. In addition, the 17 SNPs from the CUMA assays allowed for placement of a larger panel of 340 isolates within the MIP phylogeny (Additional file 2: Table S3). Nine of these SNPs were located on the same branch as another assay, or in one instance the same branch as two other assays, so did not provide any additional genotyping information. In the final tally there were assays for eight branches of the phylogeny, with assays specific to the following prominent isolates/clades and related isolates: *B. abortus* 2308, *B. abortus* 2308 + S19, *B. melitensis* 16 M, *B. melitensis* biovar 1, and *B. suis* 1330. From our diverse isolate collection we had the following distribution of calls for the branches, with the most derived call taking precedence over more ancestral calls: A = 1, B = 23, C = 8, D = 22, E = 7, F = 0, G = 15, H = 91, I = 33, J = 17, no derived call (all isolates not in species *B. abortus*, *B. melitensis*, or *B. suis*/ *canis*) = 25, no call for any assay = 7, ancestral within *B. abortus* = 12, ancestral within *B. melitensis* = 68, ancestral within *B. suis* = 11.

## Discussion

Our assays show clear distinctions within and among *B. abortusB. melitensis*, and *B. suis*. Our CUMA assays targeted clade-specific SNPs that can be incorporated into most other genotyping assays such as TaqMan Real-time PCR for increased sensitivity [18,19]. We have identified several important targets that should prove useful for clinical, epidemiological, and forensic purposes. For example, the assays targeting branches A, D, and I are specific to isolates closely related to *B. abortus* 2308 and *B. abortus* 9–941, and *B. suis* 1330, respectively. The assays for F and G target the same branch and identify *B. melitensis* 16 M and closely related isolates. Isolates from *B. abortus* 2308 and 9–941, *B. suis* 1330, and *B. melitensis* 16 M are from common, genetically monomorphic clades of *Brucella* and the SNP assays developed here are a reliable and useful way of identifying these four common groups.

Branch E is particularly interesting in terms of *Brucella* taxonomy. The clade that this branch defines includes isolates from *B. abortus* biovars 1, 2, and 4. Potential issues with biovar and phylogenetic correspondence in *B. abortus* have been noted previously [20]. Upon closer evaluation of the whole genomes used in our analyses, the apparent paraphyly within *B. abortus* biovar 1, since isolates from biovar 2 are within the biovar 1 clade, does not hold true when all the genomes are included. However, CUMA assays indicate that at least four isolates from other *B. abortus* biovars (3 of biovar 4, 1 of biovar 2) fall onto the B/C branch. This would suggest that either biovar 1 is paraphyletic or there have been issues with biovar determination.

SNP-based approaches also enable assessment of errors in genome sequences. Whole genome comparisons of the region associated with SNP10621, which were intended to target branch J in *B. suis*/ *B. canis*, also share a SNP allele with *B. abortus* 9–941. Taken at face value, this would suggest homoplasy at this locus. Yet, in our CUMA assays *B. abortus* 9–941 did not group with *B. suis*, likely indicating sequencing error.

Finding nucleotide polymorphisms that differentiate clades, species, or isolates is dependent on the genomes used for SNP discovery. In general, one will only find those SNPs that exist among the genomic samples used in the comparisons and novel SNPs will remain undiscovered [21]. This discovery bias can strongly affect taxonomic interpretation of results [22,23]. Although discovery bias is often less consequential for genotyping efforts, the effects of our choice of strains for SNP discovery are clearly apparent in our phylogenetic tree. The discovery strains are distinguished by their positions at terminal branches in the phylogeny. There is greater diversity observed in *B. abortus* simply because two strains were part of the discovery panel. Furthermore, although isolates on a branch will be grouped by the SNPs they share (or do not share), additional structure exists in the “true” phylogeny that is not apparent in the genotype tree. Branch lengths are also highly affected by the SNP discovery process. Species that are basal within this phylogeny, such as *B. cetiB. pinnipedialisB. ovis*, and *B. neotomae* have short branch lengths merely because these genomes were not part of SNP discovery. It must also be noted that *B. suis* biovar 5 is part of this basal group. SNPs that should group it with the rest of the *B. suis* clade were not present in our MIP assay, which is not surprising since this branch is extremely short, even with whole genome analysis [JTF unpubl. data, [24]. We did not observe differentiation of these and the other *Brucella* species, nor did we expect it because genomes from these groups were not a part of SNP discovery.

Whole genome resequencing at the Broad Institute of MIT/Harvard recently generated genomes for over 100 additional *Brucella* strains and these genomes should provide a broad basis for future genotyping efforts, with canonical SNPs developed for each of the important isolates and clades. Future genotyping efforts should include SNPs from all of the recognized species and biovars. Comparative work using some of these genomes has already been fruitful, demonstrating the emergence of the marine *Brucella* from within the terrestrial *Brucella* and showing a methodology for whole genome analysis [24].

A trade-off exists in current genotyping efforts between throughput and genomic sampling. Does one aim for a maximum amount of potentially informative loci through approaches such as whole genome sequencing but having to sacrifice the number of isolates that can be evaluated? Or does one aim for more complete sampling of large numbers of isolates but with a limited set of loci using individual SNP assays such as CUMA? Of course the ultimate answer depends on your research interest or clinical application as well as the amount of resources at hand. MIP assays provide phylogenetic resolution for an intermediate number of samples and intermediate number of SNPs. Nonetheless, MIP assays, or any assays based on previously discovered SNPs, will always have their inference limited by the genomes used in SNP discovery [21]. MIP assays do however allow for a focus on resolving branches of specific interest. Data from these assays then allows for targeted down selection of loci so that focal branches and isolates on them can be thoroughly interrogated using individual SNP assays. Identifying canonical SNPs and verifying their ability to differentiate clades by screening large numbers of isolates is the essential part of genotyping [17]. Less important is the type of assay used for SNP differentiation because it is highly dependent on the numbers of SNPs and samples one wants to screen. The MIP and CUMA SNP screening techniques are just two of many methods that can be used for SNP genotyping in *Brucella* and other bacteria.

## Conclusions

We developed and evaluated two different SNP-based genotyping systems for three well studied species of *Brucella*: *B. abortus*, *B. melitensis*, and *B. suis*. The first genotyping approach, using Molecular Inversion Probes, divided the species into its three respective groups and allowed for finer scale genetic resolution. Notably, this resolution occurred almost entirely within the lineages of the four strains that were used for SNP discovery: *B. abortus* 2308, *B. abortus* 9–941, *B. melitensis* 16 M, and *B. suis* 1330. This is to be expected since the choice of genomes for SNP discovery has a pervasive effect on the phylogenetic patterns that can be determined. We followed the MIP assay with development of Capillary electrophoresis Universal-tailed Mismatch Amplification mutation assays that targeted major branch points in the MIP phylogeny. We then genotyped a large and diverse collection of isolates. The main result is the development of fine scale genotyping assays that target among the most important and widespread lineages of *Brucella*. Moreover, these and closely related isolates can be easily and quickly distinguished from all other *Brucella* isolates.

Despite the era of whole genome sequencing being upon us, SNP-based genotyping and other targeted assays will remain relevant. Sequencing technology is advancing rapidly and costs per genome are quickly diminishing such that whole genome genotyping is the future of phylogenetics, forensics, and diagnostics. In fact, whole genome genotyping will soon be cost competitive with most other genotyping strategies and will have the advantage of capturing nearly all of the genetic variation with no issues of discovery bias. Nonetheless, targeted assays will remain a viable option for such goals as rapidly and easily characterizing large strain collections, clinical samples, and samples containing only trace amounts of DNA. Concerted efforts must be made to incorporate data from earlier genotyping strategies into genomic databases so this wealth of genetic information is not lost in the rush to sequence everything.

## Methods

### SNP selection

SNPs were selected by comparisons of the four *Brucella* genomes that were available at the time of MIP development: *B. melitensis* 16 M [25], *B. suis* 1330 [13], *B. abortus* 2308 [26] and *B. abortus* 9–941 [12]. SNPs from the whole genome sequences were discovered using an in-house pipeline that performs pairwise comparisons of 200 base regions around each SNP using MUMMER [see [14]. Determining the quality of the putative SNPs is essential because only high quality sequence data should be used for developing genotyping analyses [27]. Quality measures included the number of bases between SNPs and the number of bases that are conserved on each side of a SNP within a specified region. To reduce the potential effects of sequencing error, we then incorporated sequencing quality scores from Phred values. We selected only those putative SNPs with quality scores ≥30, average quality scores of SNP flanking regions (30 base pairs) ≥ 30, and where each base in the flanking regions had a quality score ≥ 20. Perl and Java scripts were then employed for additional alignments and to compile and summarize the data. Using this process, 1000 putative SNPs were selected for interrogation by the MIP chip. SNP locations and flanking regions of 40 bases on each side were sent to the manufacturer for assay design (Affymetrix, Santa Clara, CA).

### MIP primers and probes

The MIP workflow is relatively straightforward: 1) SNPs are first discovered using comparisons of whole genomes or particular regions of interest within sequenced genomes; 2) a series of assays are created with primers targeting each SNP; 3) amplification products are generated in a single multiplexed PCR; 4) amplicons specific to each SNP for each sample are hybridized to a universal tag microarray; 5) each SNP is fluorescently labeled based on the corresponding nucleotide of the sample and is then visualized on the microarray.

Primers and probes were designed for a GeneChip Custom 5 K SNP Kit (Affymetrix), which is one of the available forms of the MIP assay. In this assay, all 1000 SNPs were assessed in a single multiplex reaction for each sample. Assays containing ~3000 *Francisella tularensis* SNPs [28] and ~1000 *Burkholderia pseudomallei* SNPs (Keim unpubl. data) were run concurrently on the same chip, which reduced the cost of the assays for each group. MIP technology involves a specific probe that binds to flanking sequence surrounding a SNP site. Due to the orientation of the oligonucleotide sequence, the probe anneals as an inverted loop and a single base gap is created at the SNP site. The base at the SNP site is then added in one of four reactions involving unlabeled nucleotides. After ligation and exonuclease steps, the probe released from the sequence is amplified with PCR using universal primers specific for a portion of all probes. Only those probes where the SNP base has been added are successfully amplified. For a full description of the MIP methodology, see Hardenbol et al. [16]. Typically, approximately 80% of the MIP probes that are designed pass quality control and assurance standards at Affymetrix. Consistent with this standard, 833 of the 1000 probes (83.3%) amplified in our panel of 85 *Brucella* isolates for at least 80% of SNP alleles at a locus. Among these SNPs, 56 were monomorphic, leaving a final set of 777 phylogenetically informative loci. This dataset contained only 4% missing data, which were given an allele of N in phylogenetic analyses. To allow this dataset to be directly comparable to SNPs from whole genome analyses, we then did an *in silico* comparison of 28 whole genome sequences of *Brucella* from GenBank (Additional file 3: Table S1). Not all of the SNPs in the final set were present in all genomes or had likely duplication events so were removed from the analysis, resulting in 735 SNPs for phylogenetic analysis.

### DNA samples

We ran 85 *Brucella* DNA samples on the MIP assay from a diverse isolate collection that included *B. abortus* (33), *B. melitensis* (30), *B. suis* (11), *B. canis* (6), *B. neotomae* (1), *B. ovis* (1), *B. ceti* (1), and *B. pinnipedialis* (2). The 85 samples tested are indicated (Additional file 4: Table S2). We focused our sampling on the first three species because SNP discovery had been conducted with the genomes of only these species and thus differentiation would be restricted primarily to these species [21,22]. Samples were analyzed at a range of concentrations, from 0.6 - 20 ng/μl. Our larger panel of isolates (*n* = 340), used only in the CUMA assays (detailed below), is from a portion of our DNA collection, which came from a variety of sources (Additional file 4: Table S2). DNA was extracted using several different methods including chloroform, kit-based, and heat soak DNA extractions, although the extraction method was not always known for each sample. Isolates were largely recent, coming from sampling in the past 15 years. We note that the majority of samples came from the United States so this collection does not represent a truly global sampling.

### Phylogenetics and CUMA assays

We created a matrix of SNP alleles for all SNP positions and formatted the data as one concatenated sequence for each sample. We analyzed this sequence in PAUP* using a heuristic search with the maximum parsimony algorithm, simple sequence addition and TBR branch swapping [29]. We rooted the phylogeny with *Brucella* sp. 83/13 because of its basal position in the *Brucella* phylogeny for the isolates in our screening panel (unpubl. data). The 83/13 isolate came from an Australian rodent and data suggest that it is related to the traditional *Brucella* spp. [30] but likely diverged from the main/core *Brucella*. Using the phylogeny developed from the MIP assay to determine groups, we employed clade-specific SNPs using CUMA [31], following mismatch amplification concepts [32,33]. Briefly, the CUMA assay exploits mismatch amplification differences during PCR amplification that generate different length fragments that are allele (i.e. SNP) specific. The amplification primers have unique tails that can subsequently bind to fluorescently labeled universal-tailed primers. These unique tails are added to the 5' end of the allelic primers during primer synthesis. We used the following sequences and dyes for the universal tails: VIC-acacgcacttgacttgtcttc, FAM6-acccaactgaatagagagc, PET-ctgtccttacctcaatctc, NED-atcgactgtgttaggtcac. Assays were based on a range of amplicon sizes without overlap (range 101–259 bp) and two different dye combinations were used to visualize the fragments that discriminated each SNP state (VIC/FAM, NED/PET) (Table 1). We ran 10 mL reactions in either singleplex or multiplex with the following mastermix: 1X PCR buffer, 2 mM MgCl_2_, 0.2 mM dNTPs (Invitrogen, Carlsbad, CA), 0.2 U iTaq DNA Polymerase (Bio-Rad, Hercules, CA), and molecular grade water (GIBCO, Carlsbad, CA). Reactions were multiplexed in two different PCR reactions and fragments were run in a single capillary injection on an ABI 3100 Genetic Analyzer (Applied Biosystems, Foster City, CA). Thermocycler conditions were as follows: hot start of 95°C for 5 min, followed by 40 cycles of 30 s at 95°C, 30 s at 60°C and 30 s at 72°C, with a final extension at 72°C for 5 min. Melting temperatures for the primers ranged from 57.1 to 67.0°C (mean 61.7°C).

**Table 1:** **Capillary electrophoresis Universal-tailed Mismatch Amplification mutation assays for genotyping single nucleotide polymorphisms in** ***Brucella*** **isolates**

**SNP assay**	**Allelic Primer1**	**Allelic Primer2**	**Consensus Primer**	**Amplicon Size**	**Dye Set**	**Allele**	**SNP position in 16 M**	**Branch**
2366	CACGGCCTATCTGCTGGGCT	CACGGCCTATCTGCTGGGAC	GAGCGTCGTGAAGTCGGTTAC	151	NED/PET	T/C	1173417	A
4748	ACAGTCAGACAAGGACCGGA	AACAGTCAGACAAGGACCGAC	GTAACAAGAACACGGCCTTTACGC	155	FAM/VIC	C/A	1744207	B
1562	CACGCAAAATAGCTAAATGAAATATAC	ACGCAAAATAGCTAAATGAAATATTG	GATGGCTTTCCGGGGCTATC	189	NED/PET	C/G	1405281	B
2922	TAAAGACGGCGATTACCGAG	GTAAAGACGGCGATTACCGTA	GACAACGCCAACGGCATTCTT	179-180	NED/PET	G/A	413931	C
991	CAGTATGAAGCTTATTTTAAGCCA	GCAGTATGAAGCTTATTTTAAGCAG	GTATGCTCAAGCGCCAAGCTG	195	FAM/VIC	G/A	1601481	C
3740	CGGAATACGAAAACTCACATTATAG	CGGAATACGAAAACTCACATTATTA	GCGGGGCCATAGGGAAATAC	133	VIC/FAM	G/A	675905	D
1344	ACACGGTTGGAATTATCCACT	ACACGGTTGGAATTATCCATC	GACCGGCAAGCTTGAATCG	171	FAM/VIC	C/T	1392400	D
5754	GCTGGAACATATAGAAAAGATCATAAAAG	GCTGGAACATATAGAAAAGATCATAAATA	GCAGCCTTCCAAGGAAAAGAACG	117	VIC/FAM	G/A	1083478	E
1522	GGTGAACATTTCGCCATCAG	GGTGAACATTTCGCCATCTA	GTTCGATGAACCTCGTGGCATT	123-124	NED/PET	G/A	497534	E
6214	ATTGAATGATGAGCGATATTGTG	TGAATGATGAGCGATATTGCA	GAGCGCTTGTCGGAGGTTGTT	110-112	PET/NED	A/G	242224	F
2995	CGAAACAGCTGAGAAGATCGAG	AAACAGCTGAGAAGATCGGC	GTTAGAAGCCTGGCCCGTTCTC	101	VIC/FAM	G/C	478183	G
8872	GCATCGAACTCATTCTCGCT	GCATCGAACTCATTCTCGTC	GCGAAATCAAGGCCCCATTTG	160	PET/NED	C/T	1170581	G
1688	CGATCTGCCAGTTGACGAGA	CGATCTGCCAGTTGACGATT	GTGCAACGCCTCACGCATAAT	181	FAM/VIC	T/A	1348434	H
5362	ATTACCACGCACCGATGAGA	ATTACCACGCACCGATGAAG	GCATCCATGACGGCGTGAAAC	240	FAM/VIC	G/A	507275	I
8306	CACGGTTGCATGGTTTGTATATA	ACGGTTGCATGGTTTGTATAAG	GAGCACCAAACCGGGTGATGT	259	FAM/VIC	G/A	1876820	I
10621	TATGCAATTCGTGTCGCATG	TATGCAATTCGTGTCGCAGA	GTTTCAGGACTTTTGGGAACTGACC	233	NED/PET	G/A	1207543	J
10621R	GGTAATTTTTCCGCTTGCGT	CGGTAATTTTTCCGCTTGCTC	GCACGGGCGCAGGCTCTTAT	250	PET/NED	C/T	1207539	J

First allele is the ancestral SNP state and has a VIC or NED dye; second allele is derived and has a FAM or PET dye. Universal tails were added to the 5' end of the allelic primers during primer synthesis. See Figures 1 and S1 for branch location of SNPs in phylogeny. SNP positions are given for *B. melitensis* 16 M genome and all are on chromosome I except assays 6214 and 2995 are on chromosome II.

SNPs used in the CUMA were randomly selected from the various options available on each branch, with fewer options possible with shorter branches. If development of the assay failed to produce effective primer pairs based on standard primer design parameters we simply selected a new SNP locus. Using the CUMA assays, we genotyped a diverse set of isolates (*n* = 340), which included all recognized biovars and type strains (except *B. microti* and *B. suis* biovars 3 and 5), against 17 SNP assays for 10 branches. For each sample we determined if the SNP allele for each locus was ancestral or derived on the corresponding branch and then verified where the sample was placed on the tree. When possible, we selected two SNPs from each of the major branches. We generated amplicons for the SNP regions in four PCR reactions for each of the two multiplex PCRs and then pooled the PCR product in one capillary injection. If the CUMA assay failed any locus in multiplex reactions, we reran that locus in singleplex, which generally allowed for determination of the SNP allele. Samples with singleplex failure largely appeared to be of poor DNA quality since there were typically failures across several different CUMA assays (Additional file 4: Table S2).

## Authors’ Contributions

JTF wrote the manuscript and performed data analysis; LBP carried out molecular genetic analysis and helped draft the manuscript; WDB, DMK, CAA, and CML performed molecular genetic analysis for MIPs and CUMA; SMBS, TP, and JBS carried out data analysis; FFR provided strains, data analysis and helped draft the manuscript; PK conceived of the study, participated in its design, and helped draft the manuscript. All authors read and approved the final manuscript.

## Supplementary Material

Additional file 1 Figure S1. ***Brucella*phylogeny using maximum parsimony developed using 777 single nucleotide polymorphisms.** Letters on branches refer to phylogenetic locations of CUMA assays developed in this work. Stars on branches represent phylogenetic locations of species or clade specific assays from Foster et al. 2008. In this figure we rooted with *B. neotomae* because it is the most basal taxon in the *Brucella* phylogeny for these taxa tested (unpubl. data). (PDF 284 kb). Click here for file

Additional file 2 Table S3. **List of *Brucella*DNA samples tested with CUMA.** DNA samples came from the following institutions, Louisiana State University (LSU), California Department of Health Services (CDHS), U.S. Armed Forces Institute of Pathology (AFIP), Alaska Public Health Laboratory (APHL), Brigham Young University (BYU), U.S. Centers for Disease Control (CDC), USDA-National Animal Disease Center (NADC), and the Arizona Department of Health Services (ADHS). Samples with a species name in the branch column were genotyped as that species using assays in (Foster et al. 2008) but gave all ancestral SNP alleles in our assays. Assays for *B. abortus* in blue *B. melitensis* in pink, and *B. suis/canis* in green, which correspond to the branches in Figure 1. The 85 samples also run in the MIP assay have an asterisk, except for 3 samples not run on CUMA. Samples likely mislabeled, due to incorrect branch assignment based on species/biovar, are highlighted in yellow. (PDF 135 kb). Click here for file

Additional file 3 Table S1. **List of 28 whole genomes used for*in silico*comparisons to SNP alleles from MIP assay.** (PDF 62 kb). Click here for file

Additional file 4 Table S2. **List of*Brucella*isolates used in 17 CUMA assays, including isolate name, species, and biovar when known or applicable and the SNP allele for each assay.** (PDF 44 kb). Click here for file
