# Supplementary figures and images for: Genotyping of Brucella species using clade specific SNPs
Source: BMC Microbiol. 2012 Jun 19;12:110. doi: 10.1186/1471-2180-12-110 (PMC3747857; doi:10.1186/1471-2180-12-110)

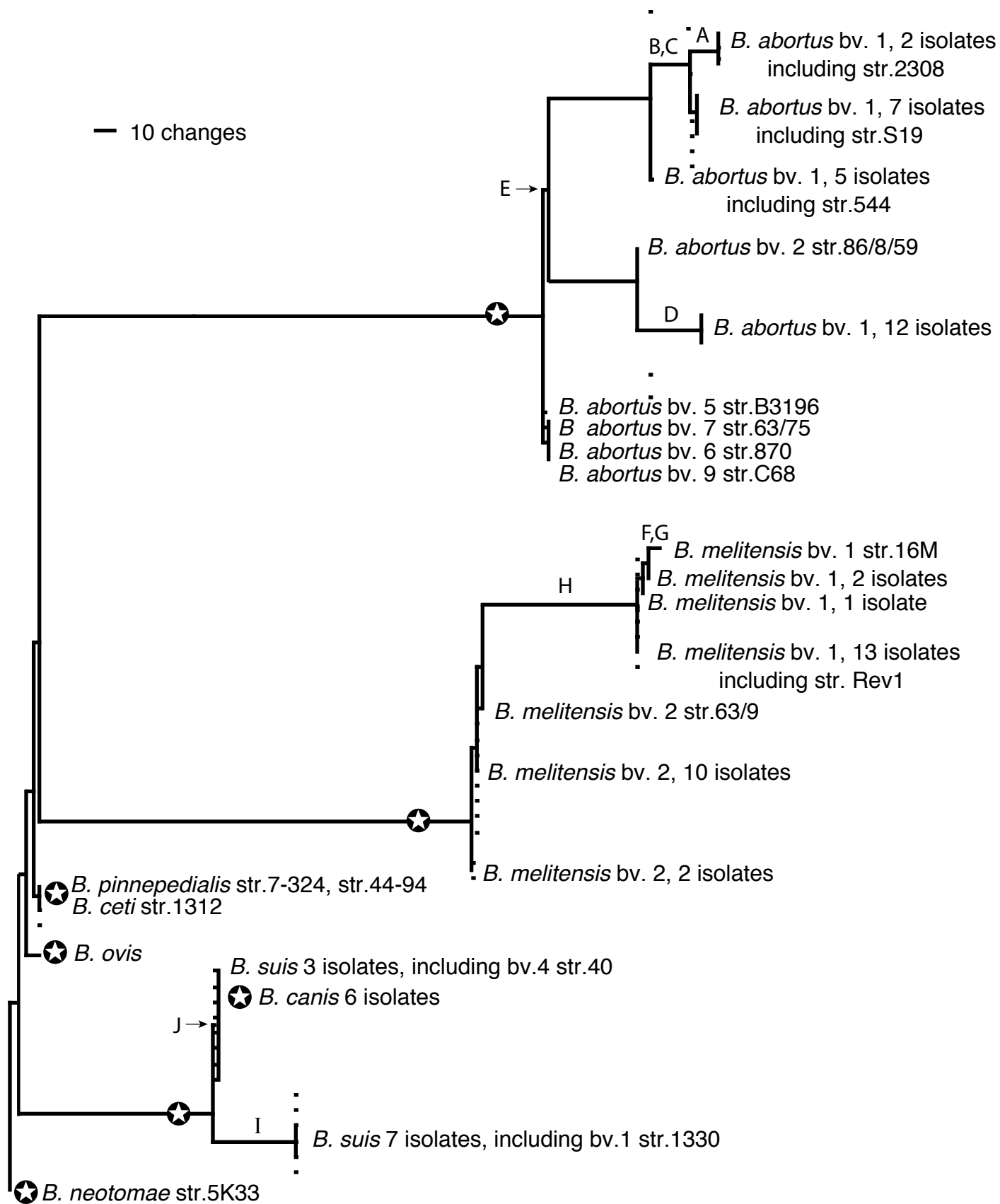

Supplement: Additional file 1 Figure S1. — Brucellaphylogeny using maximum parsimony developed using 777 single nucleotide polymorphisms. Letters on branches refer to phylogenetic locations of CUMA assays developed in this work. Stars on branches represent phylogenetic locations of species or clade specific assays from Foster et al. 2008. In this figure we rooted with B. neotomae because it is the most basal taxon in the Brucella phylogeny for these taxa tested (unpubl. data). (PDF 284 kb). [file 1471-2180-12-110-S1.pdf]
