# Supplementary material for: Genotyping of Brucella species using clade specific SNPs
Source: BMC Microbiol. 2012 Jun 19;12:110. doi: 10.1186/1471-2180-12-110 (PMC3747857; doi:10.1186/1471-2180-12-110)
Supplement: Additional file 2 Table S3. — List of BrucellaDNA samples tested with CUMA. DNA samples came from the following institutions, Louisiana State University (LSU), California Department of Health Services (CDHS), U.S. Armed Forces Institute of Pathology (AFIP), Alaska Public Health Laboratory (APHL), Brigham Young University (BYU), U.S. Centers for Disease Control (CDC), USDA-National Animal Disease Center (NADC), and the Arizona Department of Health Services (ADHS). Samples with a species name in the branch column were genotyped as that species using assays in (Foster et al. 2008) but gave all ancestral SNP alleles in our assays. Assays for B. abortus in blue B. melitensis in pink, and B. suis/canis in green, which correspond to the branches in Figure 1. The 85 samples also run in the MIP assay have an asterisk, except for 3 samples not run on CUMA. Samples likely mislabeled, due to incorrect branch assignment based on species/biovar, are highlighted in yellow. (PDF 135 kb). [file 1471-2180-12-110-S2.pdf]

Additional file 4: Table S3. List of *Brucella* DNA samples tested with CUMA. DNA samples came from the following institutions, Louisiana State University (LSU), California Department of Health Services (CDHS), U.S. Armed Forces Institute of Pathology (AFIP), Alaska Public Health Laboratory (APHL), Brigham Young University (BYU), U.S. Centers for Disease Control (CDC), USDA-National Animal Disease Center (NADC), and the Arizona Department of Health Services (ADHS). Samples with a species name in the branch column were genotyped as that species using assays in (Foster et al. 2008) but gave all ancestral SNP alleles in our assays. Assays for *B. abortus* in blue *B. melitensis* in pink, and *B. suis/canis* in green, which correspond to the branches in Fig. 1. The 85 samples also run in the MIP assay have an asterisk, except for 3 samples not run on CUMA. Samples likely mislabeled, due to incorrect branch assignment based on species/biovar, are highlighted in yellow.

| **Sample name** | **Species** | **Biovar** | **2366 A** | **1562 B** | **4748 B** | **2922 C** | **991 C** | **1344 D** | **3740 D** | **1522 E** | **5754 E** | **6214 F** | **2995RC G** | **8872 G** | **1688 H** | **5362 I** | **8306 I** | **10621 J** | **10621RC J** | **Branch** | **Collection Locale** | **Originating institution** |
|---|---|---|---|---|---|---|---|---|---|---|---|---|---|---|---|---|---|---|---|---|---|
| 2308* | *B. abortus* | 1 | 1 | 1 | 1 | 1 | 1 | 0 | 0 | 1 | 1 | 0 | 0 | 0 | 0 | 0 | 0 | 0 | 0 | A | --- | LSU |
| 2308MR | *B. abortus* | 1 | 0 | 1 | 1 | 1 | 1 | 0 | 0 | 1 | 1 | 0 | 0 | 0 | 0 | 0 | 0 | 0 | 0 | B | --- | LSU |
| PHE1 | *B. abortus* | 1 | 0 | 1 | 1 | 1 | 1 | 0 | 0 | 1 | 1 | 0 | 0 | 0 | 0 | 0 | 0 | - | 0 | B | --- | LSU |
| S19* | *B. abortus* | 1 | 0 | 1 | 1 | - | 1 | 0 | 0 | 1 | 1 | 0 | 0 | 0 | 0 | 0 | 0 | - | 0 | B | --- | LSU |
| B.canis | *B. canis* | - | 0 | 0 | 0 | 0 | 0 | 0 | 0 | 0 | 0 | 0 | 0 | 0 | 0 | 0 | 0 | 1 | 1 | J | --- | LSU |
| REV-1* | *B. melitensis* | 1 | 0 | 0 | 0 | 0 | 0 | 0 | 0 | 0 | 0 | 0 | 0 | 0 | 1 | 0 | 0 | - | 0 | H | --- | LSU |
| B.neotomae | *B. neotomae* | - | 0 | 0 | 0 | 0 | 0 | 0 | 0 | 0 | 0 | 0 | 0 | 0 | 0 | 0 | 0 | 0 | 0 | no derived | --- | LSU |
| LSU1* | *B. melitensis* | - | 0 | 0 | 0 | 0 | 0 | 0 | 0 | 0 | 0 | 0 | 0 | 0 | 1 | 0 | 0 | 0 | 0 | H | --- | LSU |
| LSU2 | *B. melitensis* | - | 0 | 0 | 0 | 0 | 0 | 0 | 0 | 0 | 0 | 0 | 0 | 0 | 1 | - | 0 | 0 | 0 | H | --- | LSU |
| LSU3 | *B. melitensis* | - | 0 | 0 | 0 | 0 | 0 | 0 | 0 | 0 | 0 | 0 | 0 | 0 | 1 | 0 | 0 | 0 | 0 | H | --- | LSU |
| LSU4 | *B. melitensis* | - | 0 | 0 | 0 | 0 | 0 | 0 | 0 | 0 | 0 | 0 | 0 | 0 | 1 | 0 | 0 | 0 | 0 | H | --- | LSU |
| 96A3121 | *B. suis* | - | 0 | 0 | - | 0 | 0 | 0 | 0 | 0 | 0 | 0 | 0 | 0 | 0 | 1 | 1 | - | 0 | I | CA, USA | CDHS |
| 90A6673 | *B. suis* | - | 0 | 0 | - | 0 | 0 | 0 | - | 0 | 0 | 0 | 0 | 0 | 0 | 1 | 1 | 0 | 0 | I | CA, USA | CDHS |
| 90A6672 | *B. suis* | - | 0 | 0 | 0 | 0 | - | 0 | - | 0 | 0 | 0 | 0 | 0 | 0 | 1 | 1 | 0 | 0 | I | CA, USA | CDHS |
| 88A3664 | *B. suis* | - | 0 | 0 | 0 | 0 | - | 0 | - | 0 | 0 | 0 | 0 | 0 | 0 | 1 | - | 0 | 0 | I | CA, USA | CDHS |
| 84A7314 | *B. suis* | - | 0 | 0 | 0 | 0 | 0 | 0 | - | 0 | 0 | 0 | 0 | 0 | 0 | 0 | 0 | 0 | 0 | suis | CA, USA | CDHS |
| 85A402 | *B. suis* | - | 0 | 0 | - | 0 | 0 | 0 | 0 | 0 | 0 | 0 | 0 | 0 | 0 | 0 | 0 | 0 | 0 | suis | CA, USA | CDHS |
| 81A733 | *B. suis* | - | 0 | 0 | - | 0 | 0 | 0 | 0 | 0 | 0 | 0 | 0 | 0 | 0 | 1 | - | 0 | 0 | I | CA, USA | CDHS |
| 7335-1-74 | *B. suis* | - | 0 | 0 | - | 0 | 0 | 0 | - | 0 | 0 | 0 | 0 | 0 | 0 | 1 | 1 | 0 | 0 | I | CA, USA | CDHS |
| 85A1413 | *B. abortus* | - | 0 | 1 | - | - | 1 | 0 | 0 | - | - | 0 | 0 | 0 | 0 | 0 | - | 0 | 0 | B | CA, USA | CDHS |
| 85A2208 | *B. abortus* | - | 0 | 0 | 0 | 0 | - | 1 | 1 | | | 0 | 0 | 0 | 0 | 0 | - | 0 | 0 | D | CA, USA | CDHS |
| 94A5678 | *B. abortus* | - | 0 | 0 | 0 | 0 | 0 | - | 1 | 1 | 1 | 0 | 0 | 0 | 0 | 0 | 0 | - | 0 | D | CA, USA | CDHS |
| 94A1027 | *B. abortus* | - | 0 | 0 | 0 | 0 | - | 0 | 0 | - | 1 | 0 | 0 | 0 | 0 | 0 | 0 | - | 0 | E | CA, USA | CDHS |
| 93A4387 | *B. abortus* | - | - | - | 0 | 0 | 0 | 0 | - | - | 1 | 0 | 0 | - | 0 | 0 | 0 | 0 | 0 | E | CA, USA | CDHS |
| 91A3727 | *B. abortus* | - | 0 | 0 | 0 | - | - | 1 | 1 | - | 1 | 0 | 0 | 0 | 0 | 0 | 0 | 0 | 0 | D | CA, USA | CDHS |
| 91A1150 | *B. abortus* | - | 0 | 0 | 0 | 0 | - | 0 | 0 | 1 | 1 | 0 | 0 | 0 | 0 | 0 | 0 | 0 | 0 | E | CA, USA | CDHS |
| 90A7689 | *B. abortus* | - | 0 | 0 | 0 | 0 | - | 1 | 1 | - | 1 | 0 | 0 | 0 | 0 | 0 | 0 | 0 | 0 | D | CA, USA | CDHS |
| 89A585 | *B. abortus* | - | 0 | 0 | 0 | 0 | - | 0 | 0 | - | 1 | 0 | 0 | 0 | 0 | 0 | - | 0 | 0 | E | CA, USA | CDHS |
| 87A6250 | *B. abortus* | - | 0 | 0 | 0 | 0 | 0 | 1 | 1 | - | 1 | 0 | 0 | 0 | 0 | 0 | 0 | 0 | 0 | D | CA, USA | CDHS |
| 86A4573 | *B. abortus* | - | 0 | 0 | 0 | 0 | 0 | 1 | 1 | 1 | 1 | 0 | 0 | 0 | 0 | 0 | - | 0 | 0 | D | CA, USA | CDHS |
| 92A1253 | *B. melitensis* | - | 0 | 0 | 0 | 0 | 0 | 0 | 0 | 0 | 0 | 0 | 0 | 0 | 1 | 0 | 0 | 0 | 0 | H | CA, USA | CDHS |
| 91A1655 | *B. melitensis* | - | 0 | - | 0 | 0 | 0 | 0 | - | 0 | 0 | 0 | 0 | 0 | 1 | - | 0 | 0 | 0 | H | CA, USA | CDHS |
| 85A2734 | *B. melitensis* | - | 0 | 0 | - | - | - | 0 | 0 | 0 | 0 | 0 | 1 | 1 | 1 | - | 0 | 0 | 0 | G | CA, USA | CDHS |
| 85A1309 | *B. melitensis* | - | 0 | 0 | 0 | 0 | 0 | 0 | 0 | 0 | 0 | 0 | 0 | 0 | 1 | - | 0 | - | 0 | H | CA, USA | CDHS |
| 85A548 | *B. melitensis* | - | 0 | 0 | 0 | 0 | 0 | 0 | 0 | 0 | 0 | 0 | 0 | 0 | 1 | - | 0 | 0 | 0 | H | CA, USA | CDHS |
| 83A8405 | *B. melitensis* | - | 0 | 0 | 0 | 0 | - | 0 | 0 | 0 | 0 | 0 | 0 | 0 | 1 | - | 0 | 0 | 0 | H | CA, USA | CDHS |
| 83A4984 | *B. melitensis* | - | 0 | 0 | 0 | 0 | 0 | 0 | 0 | - | - | 0 | 0 | 0 | 1 | 0 | - | 0 | 0 | H | CA, USA | CDHS |
| 82A8164 | *B. melitensis* | - | 0 | 0 | 0 | 0 | 0 | 0 | 0 | 0 | 0 | 0 | 0 | 0 | 1 | 0 | 0 | 0 | 0 | H | CA, USA | CDHS |
| 82A5405 | *B. melitensis* | - | 0 | 0 | 0 | 0 | - | 0 | - | 0 | 0 | 0 | 0 | 0 | 1 | 0 | 0 | 0 | 0 | H | CA, USA | CDHS |
| 81A7993 | *B. melitensis* | - | 0 | 0 | 0 | 0 | 0 | 0 | 0 | 0 | 0 | 0 | 0 | 0 | 1 | 0 | 0 | 0 | 0 | H | CA, USA | CDHS |
| 81A3047 | *B. melitensis* | - | 0 | 0 | 0 | 0 | - | 0 | 0 | 0 | 0 | 0 | 0 | 0 | 1 | - | 0 | 0 | 0 | H | CA, USA | CDHS |
| 82A324* | *B. canis* | - | 0 | 0 | 0 | 0 | 0 | 0 | 0 | 0 | 0 | 0 | 0 | 0 | 0 | 0 | 0 | 1 | 1 | J | CA, USA | CDHS |
| 16M* | *B. melitensis* | 1 | 0 | 0 | - | 0 | - | 0 | 0 | 0 | 0 | 0 | 0 | 0 | 0 | - | - | - | - | melitensis | --- | AFIP |
| 63/9* | *B. melitensis* | 2 | 0 | 0 | 0 | 0 | - | 0 | 0 | 0 | 0 | 0 | 0 | 0 | 0 | - | - | 0 | 0 | melitensis | --- | AFIP |
| Ether | *B. melitensis* | 3 | 0 | 0 | 0 | 0 | 0 | 0 | 0 | 0 | 0 | 0 | 0 | 0 | 1 | 0 | 0 | 0 | 0 | H | --- | AFIP |
| F3582* | *B. melitensis* | - | 0 | 0 | 0 | 0 | 0 | 0 | 0 | 0 | 0 | 0 | 0 | 0 | 1 | 0 | 0 | 0 | 0 | H | IL, USA | AFIP |
| F7137 | *B. melitensis* | - | 0 | 0 | 0 | 0 | 0 | 0 | 0 | 0 | 0 | 0 | 0 | 0 | 1 | 0 | 0 | 0 | 0 | H | IL, USA | AFIP |
| G3195 #1 | *B. melitensis* | 1 | 0 | 0 | 0 | 0 | 0 | 0 | 0 | 0 | 0 | 0 | 0 | 0 | 1 | 0 | 0 | 0 | 0 | H | IL, USA | AFIP |
| G3314* | *B. melitensis* | 1 | 0 | 0 | 0 | 0 | 0 | 0 | 0 | 0 | 0 | 0 | 0 | 0 | 1 | 0 | 0 | 0 | - | H | IL, USA | AFIP |
| G3315 | *B. melitensis* | 1 | 0 | 0 | 0 | 0 | 0 | 0 | 0 | 0 | 0 | 0 | 0 | 0 | 1 | 0 | 0 | 0 | 0 | H | IL, USA | AFIP |

| | | | | | | | | | | | | | | | | | | | | | | |
|---|---|---|---|---|---|---|---|---|---|---|---|---|---|---|---|---|---|---|---|---|---|---|
| G3339 | *B. melitensis* | 1 | 0 | 0 | 0 | 0 | 0 | 0 | 0 | 0 | 0 | 0 | 0 | 0 | 0 | 0 | 0 | 0 | 0 | melitensis | IL, USA | AFIP |
| G5013 | *B. melitensis* | 1 | 0 | 0 | 0 | 0 | 0 | 0 | 0 | 0 | 0 | 0 | 0 | 0 | 1 | 0 | 0 | 0 | 0 | H | IL, USA | AFIP |
| G5251* | *B. melitensis* | 1 | 0 | 0 | 0 | 0 | 0 | 0 | 0 | 0 | 0 | 0 | 0 | 0 | 1 | 0 | 0 | 0 | - | H | IL, USA | AFIP |
| G6315 | *B. melitensis* | 1 | 0 | 0 | 0 | 0 | 0 | 0 | 0 | 0 | 0 | 0 | 0 | 0 | 0 | 0 | 0 | 0 | 0 | melitensis | IL, USA | AFIP |
| G6528 | *B. melitensis* | - | 0 | 0 | 0 | 0 | 0 | 0 | 0 | 0 | 0 | 0 | 0 | 0 | 0 | 0 | 0 | 1 | 1 | J | IL, USA | AFIP |
| G7668 | *B. melitensis* | 1 | 0 | 0 | 0 | 0 | 0 | 0 | 0 | 0 | 0 | 0 | 0 | 0 | 1 | 0 | 0 | 0 | - | H | IL, USA | AFIP |
| G8496 | *B. melitensis* | 1 | 0 | 0 | 0 | 0 | 0 | 0 | 0 | 0 | 0 | 0 | 0 | 0 | 1 | 0 | 0 | 0 | - | H | IL, USA | AFIP |
| G9586 | *B. melitensis* | 2 | 0 | 0 | 0 | 0 | 0 | 0 | 0 | 0 | 0 | 0 | 0 | 0 | 1 | 0 | 0 | 0 | - | H | IL, USA | AFIP |
| G9589 | *B. melitensis* | 2 | 0 | 0 | 0 | 0 | 0 | 0 | 0 | 0 | 0 | 0 | 0 | 0 | 1 | 0 | 0 | 0 | - | H | IL, USA | AFIP |
| G9968* | *B. melitensis* | 1 | 0 | 0 | 0 | 0 | 0 | 0 | 0 | 0 | 0 | 0 | 0 | 0 | 1 | 0 | 0 | - | - | H | IL, USA | AFIP |
| G9969 | *B. melitensis* | 1 | 0 | 0 | 0 | 0 | 0 | 0 | 0 | 0 | 0 | 0 | 0 | 0 | 1 | 0 | 0 | 0 | 0 | H | IL, USA | AFIP |
| H0010 | *B. melitensis* | 3 | 0 | 0 | 0 | 0 | 0 | 0 | 0 | 0 | 0 | 0 | 0 | 0 | 1 | 0 | 0 | - | - | H | IL, USA | AFIP |
| H0011 | *B. melitensis* | 2 | 0 | 0 | 0 | 0 | 0 | 0 | 0 | 0 | 0 | 0 | 0 | 0 | 1 | 0 | 0 | 0 | - | H | IL, USA | AFIP |
| H0451 | *B. melitensis* | 1 | 0 | 0 | 0 | 0 | 0 | 0 | 0 | 0 | 0 | 0 | 0 | 0 | 1 | 0 | 0 | 0 | - | H | IL, USA | AFIP |
| H0769 | *B. melitensis* | 1 | 0 | 0 | 0 | 0 | 0 | 0 | 0 | 0 | 0 | 0 | 0 | 0 | 0 | 0 | 0 | 0 | - | melitensis | IL, USA | AFIP |
| H0866 | *B. melitensis* | 1 | 0 | 0 | 0 | 0 | 0 | 0 | 0 | 0 | 0 | 0 | 0 | 0 | 0 | 0 | 0 | 0 | - | melitensis | IL, USA | AFIP |
| F1850 | *B. melitensis* | 2 | 0 | 0 | 0 | 0 | 0 | 0 | 0 | 0 | 0 | 0 | 0 | 0 | 0 | 0 | - | 0 | - | melitensis | IL, USA | AFIP |
| F2225 | *B. melitensis* | - | 0 | 0 | 0 | 0 | 0 | 0 | 0 | 0 | 0 | 0 | 0 | 0 | 1 | 0 | 0 | 0 | 0 | H | --- | AFIP |
| F2952 | *B. melitensis* | - | 0 | 0 | 0 | 0 | 0 | 0 | 0 | 0 | 0 | 0 | 0 | 0 | 1 | 0 | 0 | 0 | - | H | IL, USA | AFIP |
| F6679 | *B. melitensis* | 1 | 0 | 0 | 0 | 0 | 0 | 0 | 0 | 0 | 0 | 0 | 0 | 0 | 1 | 0 | 0 | 0 | 0 | H | IL, USA | AFIP |
| F7476 | *B. melitensis* | 1 | 0 | 0 | 0 | 0 | 0 | 0 | 0 | 0 | 0 | 0 | 0 | 0 | 1 | 0 | 0 | 0 | - | H | IL, USA | AFIP |
| F8205 | *B. melitensis* | 1 | 0 | 0 | 0 | 0 | 0 | 0 | 0 | 0 | 0 | 0 | 0 | 0 | 1 | 0 | 0 | 0 | - | H | IL, USA | AFIP |
| G1937 | *B. melitensis* | 1 | 0 | 0 | 0 | 0 | 0 | 0 | 0 | 0 | 0 | 0 | 0 | 0 | 1 | 0 | 0 | 0 | 0 | H | IL, USA | AFIP |
| G2009 | *B. melitensis* | 1 | 0 | 0 | 0 | 0 | 0 | 0 | 0 | 0 | 0 | 0 | 0 | 0 | 0 | 0 | 0 | 0 | 0 | melitensis | IL, USA | AFIP |
| G6191 | *B. melitensis* | 1 | 0 | 0 | 0 | 0 | 0 | 0 | 0 | 0 | 0 | 0 | 1 | 1 | 1 | 0 | 0 | 0 | 0 | G | IL, USA | AFIP |
| G7080 | *B. melitensis* | 1 | 0 | 0 | 0 | 0 | 0 | 0 | 0 | 0 | 0 | 0 | 0 | 0 | 1 | 0 | 0 | - | 0 | H | IL, USA | AFIP |
| G8551 | *B. melitensis* | - | 0 | 0 | 0 | 0 | 0 | 0 | 0 | 0 | 0 | 0 | 0 | 0 | 1 | 0 | 0 | 0 | 0 | H | IL, USA | AFIP |
| G9560 | *B. melitensis* | 3 | 0 | 0 | 0 | 0 | 0 | 0 | 0 | 0 | 0 | 0 | 0 | 0 | 1 | 0 | 0 | 0 | 0 | H | IL, USA | AFIP |
| H0110 | *B. melitensis* | 1 | 0 | 0 | 0 | 0 | 0 | 0 | 0 | 0 | 0 | 0 | 0 | 0 | 1 | 0 | 0 | 0 | 0 | H | IL, USA | AFIP |
| H0369 | *B. melitensis* | 1 | 0 | 0 | 0 | 0 | 0 | 0 | 0 | 0 | 0 | 0 | 0 | 0 | 0 | 0 | 0 | 0 | 0 | melitensis | IL, USA | AFIP |
| H0687 | *B. melitensis* | 1 | 0 | 0 | 0 | 0 | 0 | 0 | 0 | 0 | 0 | 0 | 0 | 0 | 1 | 0 | 0 | - | - | H | IL, USA | AFIP |
| H0965 | *B. melitensis* | 1 | 0 | 0 | 0 | 0 | 0 | 0 | 0 | 0 | 0 | 0 | 0 | 0 | 1 | 0 | 0 | 0 | 0 | H | IL, USA | AFIP |
| H0986* | *B. melitensis* | 1 | 0 | 0 | 0 | 0 | 0 | 0 | 0 | 0 | 0 | 0 | 1 | 1 | 1 | 0 | 0 | 0 | - | G | IL, USA | AFIP |
| H0999 | *B. melitensis* | 1 | 0 | 0 | 0 | 0 | 0 | 0 | 0 | 0 | 0 | 0 | 0 | 0 | 1 | 0 | 0 | 0 | - | H | IL, USA | AFIP |
| H1105* | *B. melitensis* | 1 | 0 | 0 | 0 | 0 | 0 | 0 | 0 | 0 | 0 | 0 | 0 | 0 | 0 | 0 | 0 | 0 | - | melitensis | IL, USA | AFIP |
| H1300 | *B. melitensis* | 3 | 0 | 0 | 0 | 0 | 0 | 0 | 0 | 0 | 0 | 0 | 0 | 0 | 1 | 0 | 0 | - | 0 | H | IL, USA | AFIP |
| G6916 | *B. melitensis* | - | 0 | 0 | 0 | 0 | 0 | 0 | 0 | 0 | 0 | 0 | 0 | 0 | 1 | 0 | 0 | 0 | 0 | H | IL, USA | AFIP |
| G8755 | *B. melitensis* | - | 0 | 0 | 0 | 0 | 0 | 0 | 0 | 0 | 0 | 0 | 0 | 0 | 1 | 0 | 0 | 0 | - | H | IL, USA | AFIP |
| G9870 | *B. melitensis* | 1 | 0 | 0 | 0 | 0 | 0 | 0 | 0 | 0 | 0 | 0 | 0 | 0 | 1 | 0 | 0 | - | - | H | IL, USA | AFIP |
| G8027 | *B. melitensis* | 1 | 0 | 0 | 0 | 0 | 0 | 0 | 0 | 0 | 0 | 0 | 0 | 0 | 1 | 0 | 0 | 0 | 0 | H | IL, USA | AFIP |
| G5677 | *B. melitensis* | 1 | 0 | 0 | 0 | 0 | 0 | 0 | 0 | 0 | 0 | 0 | 0 | 0 | 0 | 0 | 0 | 0 | 0 | melitensis | IL, USA | AFIP |
| E8128 | *B. melitensis* | 1 | - | - | 0 | 0 | 0 | 0 | 0 | 0 | 0 | 0 | 0 | 0 | 0 | 0 | 0 | - | - | melitensis | IL, USA | AFIP |
| G9809 | *B. melitensis* | 2 | 0 | 0 | 0 | 0 | 0 | 0 | 0 | 0 | 0 | 0 | 0 | 0 | 1 | 0 | 0 | - | 0 | H | IL, USA | AFIP |
| E8129 | *B. melitensis* | 1 | 0 | - | 0 | 0 | 0 | 0 | 0 | 0 | 0 | 0 | 0 | 0 | 0 | 0 | 0 | - | 0 | melitensis | IL, USA | AFIP |
| E8127 | *B. melitensis* | 1 | 0 | - | 0 | 0 | 0 | 0 | 0 | 0 | 0 | 0 | 0 | 0 | 0 | 0 | 0 | 0 | 0 | melitensis | IL, USA | AFIP |
| E8130 | *B. melitensis* | 1 | 0 | 0 | 0 | 0 | 0 | 0 | 0 | 0 | 0 | 0 | 0 | 0 | 0 | 0 | 0 | 0 | 0 | melitensis | IL, USA | AFIP |
| G9482 | *B. melitensis* | 3 | 0 | 0 | 0 | 0 | 0 | 0 | 0 | 0 | 0 | 0 | 0 | 0 | 1 | 0 | 0 | 0 | 0 | H | IL, USA | AFIP |
| H0128 | *B. melitensis* | 1 | 0 | 0 | 0 | 0 | 0 | 0 | 0 | 0 | 0 | 0 | 0 | 0 | 1 | 0 | 0 | 0 | 0 | H | HI, USA | AFIP |
| H0077 | *B. melitensis* | 2 | 0 | - | - | - | - | 0 | 0 | 0 | - | 0 | 0 | 0 | - | 0 | 0 | - | - | no call | IL, USA | AFIP |
| G5353 | *B. melitensis* | 3 | 0 | 0 | 0 | 0 | 0 | 0 | 0 | 0 | 0 | 0 | 0 | 0 | 0 | 0 | 0 | 0 | 0 | melitensis | IL, USA | AFIP |
| E8117 | *B. melitensis* | 2 | 0 | 0 | 0 | 0 | 0 | 0 | 0 | 0 | 0 | 0 | 0 | 0 | 0 | 0 | 0 | 0 | 0 | melitensis | IL, USA | AFIP |
| H0130 | *B. melitensis* | 1 | 0 | 0 | 0 | 0 | 0 | 0 | 0 | 0 | 0 | 0 | 0 | 0 | 1 | 0 | 0 | 0 | 0 | H | IL, USA | AFIP |
| G9248* | *B. melitensis* | 3 | 0 | 0 | 0 | 0 | 0 | 0 | 0 | 0 | 0 | 0 | 0 | 0 | 1 | 0 | 0 | 0 | 0 | H | AZ, USA | AFIP |
| F4018 | *B. melitensis* | 2 | 0 | 0 | 0 | 0 | 0 | - | - | 0 | 0 | 0 | 0 | 0 | 0 | 0 | 0 | 0 | 0 | melitensis | Saudi Arabia | AFIP |
| G9879 | *B. melitensis* | - | 0 | 0 | 0 | 0 | 0 | 0 | 0 | 0 | 0 | 0 | 0 | 0 | 1 | 0 | 0 | 0 | 0 | H | IL, USA | AFIP |
| H1679 | *B. melitensis* | 1 | 0 | 0 | 0 | 0 | 0 | 0 | 0 | 0 | 0 | 0 | 0 | 0 | 0 | 0 | 0 | 0 | 0 | melitensis | OH, USA | AFIP |
| H1962 | *B. melitensis* | 2 | 0 | 0 | 0 | 0 | 0 | 0 | 0 | 0 | 0 | 0 | 1 | 1 | 1 | 0 | 0 | 0 | 0 | G | UT, USA | AFIP |
| H1982* | *B. melitensis* | 2 | 0 | 0 | 0 | - | - | - | - | 0 | 0 | 0 | 0 | 0 | 1 | 0 | 0 | - | - | H | --- | AFIP |
| H1992 | *B. melitensis* | 2 | 0 | 0 | 0 | 0 | 0 | 0 | 0 | 0 | 0 | 0 | 0 | 0 | 1 | 0 | 0 | 0 | 0 | H | MO, USA | AFIP |
| H2011* | *B. melitensis* | 3 | 0 | 0 | 0 | 0 | 0 | 0 | 0 | 0 | 0 | 0 | 0 | 0 | 1 | 0 | 0 | 0 | 0 | H | AR, USA | AFIP |
| H2019 | *B. melitensis* | 1 | 0 | 0 | 0 | 0 | 0 | 0 | 0 | 0 | 0 | 0 | 0 | 0 | 1 | 0 | 0 | 0 | 0 | H | MO, USA | AFIP |
| 40* | *B. suis* | 4 | 0 | 0 | - | 0 | 0 | 0 | 0 | 0 | 0 | 0 | 0 | 0 | 0 | 1 | 1 | 0 | 0 | I | --- | AFIP |
| G8920* | *B. suis* | 1 | 0 | 0 | - | 0 | 0 | 0 | 0 | 0 | 0 | 0 | 0 | 0 | 0 | 0 | 0 | 0 | 0 | suis | IL, USA | AFIP |
| G8756 | *B. suis* | 1 | 0 | 0 | - | 0 | 0 | 0 | 0 | 0 | 0 | 0 | 0 | 0 | 0 | 1 | 1 | 0 | 0 | I | IL, USA | AFIP |
| F0531 | *B. suis* | 1 | 0 | 0 | - | 0 | 0 | 0 | 0 | 0 | 0 | 0 | 0 | 0 | 0 | 1 | 1 | 0 | 0 | I | IL, USA | AFIP |
| F1183 | *B. suis* | 1 | 0 | 0 | 0 | 0 | 0 | 0 | 0 | 0 | 0 | 0 | 0 | 0 | 0 | 1 | 1 | 0 | 0 | I | FL, USA | AFIP |
| F1277A | *B. suis* | 1 | 0 | 0 | - | 0 | 0 | 0 | 0 | 0 | 0 | 0 | 0 | 0 | 0 | - | 1 | - | - | I | DL, USA | AFIP |
| F1277B | *B. suis* | 1 | 0 | 0 | - | 0 | 0 | 0 | 0 | 0 | 0 | 0 | 0 | 0 | 0 | 0 | - | 0 | 0 | suis | DL, USA | AFIP |
| F1651A | *B. suis* | 1 | 0 | 0 | - | 0 | 0 | 0 | 0 | 0 | 0 | 0 | 0 | 0 | 0 | 1 | 1 | 0 | 0 | I | GA, USA | AFIP |
| F1651B | *B. suis* | 1 | 0 | 0 | 0 | 0 | 0 | 0 | 0 | 0 | 0 | 0 | 0 | 0 | 0 | 1 | 1 | 0 | 0 | I | GA, USA | AFIP |
| F4390* | *B. suis* | 1 | 0 | 0 | - | 0 | 0 | 0 | 0 | 0 | 0 | 0 | 0 | 0 | 0 | 1 | 1 | 0 | 0 | I | LA, USA | AFIP |

| | | | | | | | | | | | | | | | | | | | | | |
|---|---|---|---|---|---|---|---|---|---|---|---|---|---|---|---|---|---|---|---|---|---|
| F4560A | *B. suis* | - | 0 | 0 | - | 0 | 0 | 0 | 0 | 0 | 0 | 0 | 0 | 0 | 0 | 1 | 1 | 0 | 0 | I | GA, USA | AFIP |
| F4560B | *B. suis* | - | 0 | 0 | - | 0 | 0 | 0 | 0 | 0 | 0 | 0 | 0 | 0 | 0 | 1 | 1 | 0 | 0 | I | GA, USA | AFIP |
| G4520 | *B. suis* | - | 0 | 0 | - | 0 | 0 | 0 | 0 | 0 | 0 | 0 | 0 | 0 | 0 | 1 | 1 | 0 | 0 | I | FL, USA | AFIP |
| G6656 | *B. suis* | 1 | 0 | 0 | 0 | 0 | 0 | 0 | 0 | 0 | 0 | 0 | 0 | 0 | 0 | 0 | 0 | 0 | 0 | suis | GA, USA | AFIP |
| G7644 | *B. suis* | - | 0 | 0 | - | 0 | 0 | 0 | 0 | 0 | 0 | 0 | 0 | 0 | 0 | - | - | 0 | 0 | no call | GA, USA | AFIP |
| G9049A | *B. suis* | 1 | 0 | 0 | - | 0 | 0 | 0 | 0 | 0 | 0 | 0 | 0 | 0 | 0 | 1 | 1 | 0 | 0 | I | GA, USA | AFIP |
| G9049B | *B. suis* | 1 | 0 | 0 | - | 0 | 0 | 0 | 0 | 0 | 0 | 0 | 0 | 0 | 0 | - | - | 0 | 0 | no call | GA, USA | AFIP |
| G9880 | *B. suis* | 1 | 0 | 0 | - | 0 | 0 | 0 | 0 | 0 | 0 | 0 | 0 | 0 | 0 | 1 | - | 0 | 0 | I | GA, USA | AFIP |
| G9949 | *B. suis* | 1 | 0 | 0 | - | 0 | 0 | 0 | 0 | 0 | 0 | 0 | 0 | 0 | 0 | 1 | 1 | 0 | 0 | I | NC, USA | AFIP |
| H0117* | *B. suis* | 1 | 0 | 0 | 0 | 0 | 0 | 0 | 0 | 0 | 0 | 0 | 0 | 0 | 0 | 1 | 1 | 0 | 0 | I | HI, USA | AFIP |
| H0134A | *B. suis* | 1 | 0 | 0 | - | 0 | 0 | 0 | 0 | 0 | 0 | 0 | 0 | 0 | 0 | 1 | - | 0 | 0 | I | GA, USA | AFIP |
| H0134B | *B. suis* | 1 | 0 | 0 | 0 | 0 | 0 | 0 | 0 | 0 | 0 | 0 | 0 | 0 | 0 | - | - | 0 | 0 | no call | GA, USA | AFIP |
| 544* | *B. abortus* | 1 | 0 | 0 | 0 | | 1 | 0 | 0 | 1 | 1 | 0 | 0 | 0 | 0 | - | - | 0 | 0 | C | --- | AFIP |
| 86/8/59* | *B. abortus* | 2 | 0 | 0 | 0 | 0 | - | 0 | 0 | 1 | 1 | 0 | 0 | 0 | 0 | 0 | 0 | 0 | 0 | E | --- | AFIP |
| 292 | *B. abortus* | 4 | 0 | 0 | 0 | 0 | 0 | 0 | 0 | 0 | 0 | 0 | 0 | 0 | 0 | 0 | 0 | 0 | 0 | abortus | --- | AFIP |
| B3196* | *B. abortus* | 5 | 0 | 0 | 0 | 0 | 0 | - | 0 | 0 | 0 | 0 | 0 | 0 | 0 | 0 | 0 | - | 0 | abortus | --- | AFIP |
| 870* | *B. abortus* | 6 | 0 | 0 | 0 | 0 | 0 | 0 | 0 | 0 | 0 | 0 | 0 | 0 | 0 | 0 | 0 | 0 | 0 | abortus | --- | AFIP |
| 63/75* | *B. abortus* | 7 | 0 | 0 | 0 | 0 | 0 | 0 | 0 | 0 | 0 | 0 | 0 | 0 | 0 | 0 | 0 | 0 | 0 | abortus | --- | AFIP |
| C68* | *B. abortus* | 9 | 0 | 0 | 0 | 0 | 0 | - | 0 | 0 | 0 | 0 | 0 | 0 | 0 | 0 | 0 | 0 | 0 | abortus | --- | AFIP |
| F4552 | *B. abortus* | - | 0 | 0 | 0 | 0 | 0 | 0 | 0 | 0 | 0 | 0 | 0 | 0 | 0 | 0 | 0 | 0 | 0 | abortus | IL, USA | AFIP |
| F4902* | *B. abortus* | - | 0 | 0 | 0 | 0 | 0 | - | - | 0 | 0 | 0 | 0 | 0 | 0 | 0 | 0 | 0 | 0 | abortus | IL, USA | AFIP |
| RM-6/66 | *B. canis* | - | 0 | 0 | 0 | 0 | 0 | 0 | - | 0 | 0 | 0 | 0 | 0 | 0 | 0 | 0 | 1 | 1 | J | --- | AFIP |
| 63/290 | *B. ovis* | - | 0 | 0 | 0 | 0 | 0 | 0 | 0 | 0 | 0 | 0 | 0 | 0 | 0 | 0 | 0 | - | - | no derived | --- | AFIP |
| 5K33 | *B. neotomae* | - | 0 | 0 | 0 | 0 | 0 | 0 | 0 | 0 | 0 | 0 | 0 | 0 | 0 | 0 | 0 | 0 | 0 | no derived | --- | AFIP |
| H0877 | *B. melitensis* | 1 | 0 | - | 0 | - | - | - | - | 0 | 0 | 0 | 0 | - | 1 | - | - | - | - | H | CA, USA | AFIP |
| A8915 | *B. melitensis* | 3 | 0 | 0 | 0 | 0 | 0 | 0 | 0 | 0 | 0 | 0 | 0 | 0 | 1 | 0 | 0 | 0 | 0 | H | NM, USA | AFIP |
| G1374 | *B. melitensis* | 3 | 0 | 0 | 0 | 0 | 0 | - | - | 0 | 0 | 0 | - | 0 | 1 | 0 | 0 | - | - | H | AZ, USA | AFIP |
| G7402 | *B. melitensis* | 1 | 0 | 0 | 0 | 0 | 0 | - | - | 0 | 0 | 0 | 0 | 0 | 1 | 0 | 0 | - | 0 | H | AZ, USA | AFIP |
| F8672* | *B. canis* | - | 0 | 0 | 0 | 0 | 0 | 0 | 0 | 0 | 0 | 0 | 0 | 0 | 0 | 0 | 0 | 1 | 1 | J | NM, USA | AFIP |
| G8963 | *B. melitensis* | 3 | 0 | 0 | 0 | 0 | 0 | 0 | 0 | 0 | 0 | 0 | 0 | 0 | 0 | 0 | 0 | - | - | melitensis | AZ, USA | AFIP |
| G4087 | *B. melitensis* | - | - | - | 0 | - | - | - | - | 0 | - | - | 0 | - | 1 | - | - | - | - | H | --- | AFIP |
| G9088 | *B. melitensis* | - | 0 | 0 | 0 | 0 | 0 | 0 | 0 | 0 | 0 | 0 | 0 | 0 | 1 | 0 | 0 | 0 | 0 | H | NC, USA | AFIP |
| G9272 | *B. melitensis* | 1 | 0 | 0 | 0 | 0 | 0 | 0 | 0 | 0 | 0 | 0 | 0 | 0 | 0 | 0 | 0 | 0 | 0 | melitensis | AZ, USA | AFIP |
| F1063 | *B. abortus* | - | 0 | 0 | 0 | 0 | - | - | - | 0 | - | 0 | 0 | 0 | 0 | - | - | - | - | abortus | NV, USA | AFIP |
| F4849 | *B. melitensis* | - | 0 | 0 | 0 | 0 | 0 | - | - | 0 | - | 0 | 0 | 0 | 0 | 0 | 0 | - | - | melitensis | NM, USA | AFIP |
| H1090 | *B. abortus* | 1 | 0 | 0 | 0 | 0 | 0 | 0 | 0 | 0 | 0 | 0 | 0 | 0 | 0 | 0 | 0 | 0 | 0 | abortus | NM, USA | AFIP |
| H1224 | *B. melitensis* | 1 | 0 | 0 | 0 | 0 | - | 0 | 0 | 0 | 0 | 0 | 0 | 0 | 0 | 0 | 0 | 0 | 0 | melitensis | CA, USA | AFIP |
| H0852 | *B. melitensis* | 1 | 0 | 0 | 0 | 0 | 0 | 0 | 0 | 0 | 0 | 0 | 0 | 0 | 1 | 0 | 0 | 0 | 0 | H | MN, USA | AFIP |
| G6596 | *B. melitensis* | 1 | 0 | 0 | 0 | 0 | 0 | 0 | 0 | 0 | 0 | 0 | 1 | 1 | 1 | 0 | 0 | 0 | 0 | G | CA, USA | AFIP |
| G9535 | *B. melitensis* | 1 | 0 | 0 | 0 | 0 | 0 | 0 | 0 | 0 | 0 | 0 | 0 | 0 | 1 | - | 0 | 0 | 0 | H | NM, USA | AFIP |
| G9226 | *B. melitensis* | 3 | 0 | 0 | 0 | 0 | 0 | 0 | 0 | 0 | 0 | 0 | 1 | 1 | 1 | 0 | 0 | 0 | 0 | G | AZ, USA | AFIP |
| G8962 | *B. melitensis* | 3 | 0 | 0 | 0 | - | - | - | - | 0 | - | 0 | 0 | 0 | 0 | - | - | - | - | melitensis | IL, USA | AFIP |
| E9296 | *B. melitensis* | 3 | 0 | 0 | 0 | 0 | 0 | 0 | 0 | 0 | 0 | 0 | 1 | 1 | 1 | 0 | 0 | - | - | G | AZ, USA | AFIP |
| E7439 | *B. melitensis* | - | 0 | 0 | 0 | 0 | 0 | 0 | 0 | 0 | 0 | 0 | 0 | 0 | 1 | 0 | 0 | 0 | 0 | H | AZ, USA | AFIP |
| G9210 | *B. melitensis* | 3 | 0 | 0 | 0 | 0 | 0 | 0 | 0 | 0 | 0 | 0 | 0 | 0 | 1 | 0 | 0 | 0 | 0 | H | AZ, USA | AFIP |
| G9268* | *B. melitensis* | 3 | 0 | 0 | 0 | 0 | 0 | 0 | 0 | 0 | 0 | 0 | 0 | 0 | 1 | 0 | 0 | 0 | 0 | H | AZ, USA | AFIP |
| G9184 | *B. melitensis* | 2 | 0 | 0 | 0 | 0 | 0 | 0 | 0 | 0 | 0 | 0 | 0 | 0 | 1 | 0 | 0 | 0 | 0 | H | AZ, USA | AFIP |
| G9617 | *B. melitensis* | 1 | 0 | 0 | 0 | 0 | 0 | 0 | 0 | 0 | 0 | 0 | 0 | 0 | 1 | 0 | 0 | 0 | 0 | H | NM, USA | AFIP |
| G9249 | *B. melitensis* | 3 | 0 | 0 | 0 | 0 | 0 | 0 | 0 | 0 | 0 | 0 | 1 | 1 | 1 | 0 | 0 | 0 | 0 | G | AZ, USA | AFIP |
| G9193 | *B. melitensis* | 3 | 0 | 0 | 0 | 0 | 0 | 0 | 0 | 0 | 0 | 0 | 0 | 0 | 1 | 0 | 0 | 0 | 0 | H | AZ, USA | AFIP |
| G1688 | *B. melitensis* | - | 0 | 0 | 0 | 0 | - | - | - | 0 | 0 | 0 | 0 | 0 | 1 | 0 | 0 | - | - | H | NM, USA | AFIP |
| G9295 | *B. abortus* | 3 | 0 | 0 | 0 | 0 | 0 | 0 | 0 | 0 | 0 | 0 | 0 | 0 | 1 | 0 | 0 | - | 0 | H | AZ, USA | AFIP |
| F7914 | *B. melitensis* | - | 0 | 0 | 0 | 0 | 0 | - | - | 0 | 0 | 0 | 0 | 0 | 0 | 0 | 0 | 0 | 0 | melitensis | IL, USA | AFIP |
| G9481 | *B. melitensis* | 3 | 0 | 0 | 0 | 0 | 0 | 0 | 0 | 0 | 0 | 0 | 0 | 0 | 1 | 0 | 0 | 0 | 0 | H | AZ, USA | AFIP |
| E7479 | *B. melitensis* | 3 | 0 | 0 | 0 | 0 | 0 | 0 | 0 | 0 | 0 | 0 | 1 | 1 | 1 | 0 | 0 | 0 | 0 | G | CA, USA | AFIP |
| G7367 | *B. melitensis* | - | 0 | 0 | 0 | 0 | 0 | 0 | 0 | 0 | 0 | 0 | 0 | 0 | 0 | 0 | 0 | 0 | 0 | melitensis | CA, USA | AFIP |
| G9319 | *B. melitensis* | 3 | 0 | 0 | 0 | 0 | 0 | 0 | 0 | 0 | 0 | 0 | 0 | 0 | 1 | 0 | 0 | 0 | 0 | H | NM, USA | AFIP |
| MEX349* | *B. melitensis* | - | 0 | 0 | 0 | 0 | 0 | 0 | 0 | 0 | 0 | 0 | 0 | 0 | 1 | 0 | 0 | 0 | 0 | H | --- | AFIP |
| MEX350* | *B. melitensis* | - | 0 | 0 | 0 | 0 | 0 | - | - | 0 | - | 0 | 1 | 1 | 1 | 0 | 0 | - | 0 | G | --- | AFIP |
| MEX351* | *B. melitensis* | - | 0 | 0 | 0 | 0 | 0 | - | 0 | 0 | 0 | 0 | 1 | 1 | 1 | 0 | 0 | 0 | 0 | G | --- | AFIP |
| MEX352* | *B. melitensis* | - | 0 | 0 | 0 | 0 | 0 | 0 | 0 | 0 | 0 | 0 | 1 | 1 | 1 | 0 | 0 | - | - | G | --- | AFIP |
| G6605 | *B. melitensis* | 1 | 0 | 0 | 0 | 0 | 0 | 0 | 0 | 0 | 0 | 0 | 0 | 0 | 1 | 0 | 0 | 0 | 0 | H | NE, USA | AFIP |
| 991633* | *B. melitensis* | - | 0 | 0 | 0 | 0 | 0 | 0 | 0 | 0 | 0 | 0 | 0 | 0 | 0 | 0 | 0 | 0 | 0 | melitensis | --- | AFIP |
| 9916342 | *B. melitensis* | - | 0 | - | 0 | - | - | - | - | 0 | - | 0 | 0 | 0 | 0 | - | 0 | - | - | melitensis | --- | AFIP |
| 9916349 | *B. melitensis* | - | - | - | 0 | - | - | - | - | 0 | - | 0 | 0 | - | 0 | - | 0 | - | - | melitensis | --- | AFIP |
| 9916361* | *B. melitensis* | - | 0 | - | - | - | 0 | - | - | 0 | 0 | 0 | 0 | 0 | 0 | - | 0 | - | - | melitensis | --- | AFIP |
| 9916364 | *B. melitensis* | - | 0 | - | 0 | 0 | 0 | 0 | 0 | 0 | 0 | 0 | 0 | 0 | 0 | 0 | 0 | - | - | melitensis | --- | AFIP |
| 9916371* | *B. melitensis* | - | 0 | 0 | 0 | 0 | 0 | 0 | 0 | 0 | 0 | 0 | 0 | 0 | 0 | 0 | 0 | 0 | 0 | melitensis | --- | AFIP |
| 9916372 | *B. melitensis* | - | 0 | - | 0 | 0 | 0 | 0 | 0 | 0 | 0 | 0 | 0 | 0 | 0 | 0 | 0 | - | - | melitensis | --- | AFIP |
| 8 | *B. melitensis* | - | 0 | 0 | 0 | 0 | 0 | 0 | 0 | 0 | 0 | 0 | 0 | 0 | 0 | 0 | 0 | 0 | 0 | melitensis | --- | AFIP |
| 200 | *B. melitensis* | - | 0 | 0 | 0 | 0 | 0 | 0 | 0 | 0 | 0 | - | 0 | - | 0 | 0 | 0 | 0 | 0 | melitensis | --- | AFIP |

| | | | | | | | | | | | | | | | | | | | | | | |
|---|---|---|---|---|---|---|---|---|---|---|---|---|---|---|---|---|---|---|---|---|---|---|
| 2054 | *B. melitensis* | - | 0 | - | 0 | 0 | 0 | 0 | 0 | 0 | 0 | 0 | 0 | 0 | 0 | 0 | 0 | - | 0 | melitensis | --- | AFIP |
| 2310* | *B. melitensis* | - | 0 | 0 | 0 | 0 | 0 | 0 | 0 | 0 | 0 | 0 | 0 | 0 | 0 | 0 | 0 | 0 | 0 | melitensis | --- | AFIP |
| 3857 | *B. melitensis* | - | 0 | - | 0 | 0 | 0 | 0 | 0 | 0 | 0 | 0 | 0 | 0 | 0 | 0 | 0 | - | - | melitensis | --- | AFIP |
| 3859 | *B. melitensis* | - | 0 | 0 | 0 | 0 | 0 | 0 | 0 | 0 | 0 | 0 | 0 | 0 | 0 | 0 | 0 | 0 | 0 | melitensis | --- | AFIP |
| 4552 | *B. melitensis* | - | 0 | 0 | 0 | 0 | 0 | 0 | 0 | 0 | 0 | 0 | 0 | 0 | 0 | 0 | 0 | 0 | 0 | melitensis | --- | AFIP |
| 4553* | *B. melitensis* | - | 0 | - | 0 | 0 | 0 | 0 | 0 | 0 | 0 | 0 | 0 | 0 | 0 | 0 | 0 | - | 0 | melitensis | --- | AFIP |
| 378* | *B. melitensis* | - | 0 | 0 | 0 | 0 | 0 | 0 | 0 | 0 | 0 | - | 0 | - | 0 | 0 | 0 | 0 | 0 | melitensis | --- | AFIP |
| 5490 | *B. melitensis* | - | 0 | - | 0 | 0 | 0 | 0 | 0 | 0 | 0 | 0 | 0 | 0 | 0 | 0 | 0 | - | 0 | melitensis | --- | AFIP |
| 6912 | *B. melitensis* | - | 0 | 0 | 0 | 0 | 0 | 0 | 0 | 0 | 0 | 0 | 0 | 0 | 0 | 0 | 0 | 0 | 0 | melitensis | --- | AFIP |
| 6913 | *B. melitensis* | - | 0 | - | 0 | 0 | 0 | 0 | - | 0 | - | 0 | 0 | 0 | 0 | 0 | 0 | - | - | melitensis | --- | AFIP |
| 7445 | *B. melitensis* | - | 0 | 0 | 0 | 0 | 0 | 0 | 0 | 0 | 0 | 0 | 0 | 0 | 0 | 0 | 0 | 0 | 0 | melitensis | --- | AFIP |
| John David* | *B. melitensis* | - | 0 | 0 | 0 | 0 | 0 | 0 | 0 | 0 | 0 | 0 | 0 | 0 | 0 | 0 | 0 | 0 | 0 | melitensis | --- | AFIP |
| 6911 | *B. melitensis* | - | 0 | 0 | 0 | 0 | 0 | 0 | 0 | 0 | 0 | 0 | 0 | 0 | 0 | 0 | 0 | 0 | 0 | melitensis | --- | AFIP |
| 84A7314 | *B. suis* | - | 0 | 0 | 0 | 0 | 0 | 0 | 0 | 0 | 0 | 0 | 0 | 0 | 0 | 0 | 0 | 0 | 0 | suis | --- | AFIP |
| 9109 | *B. melitensis* | - | 0 | - | 0 | 0 | 0 | 0 | 0 | 0 | 0 | 0 | 0 | 0 | 0 | 0 | 0 | - | 0 | melitensis | --- | AFIP |
| 9110* | *B. melitensis* | - | 0 | 0 | 0 | 0 | 0 | 0 | 0 | 0 | 0 | 0 | 0 | 0 | 0 | 0 | 0 | 0 | 0 | melitensis | --- | AFIP |
| 9916381 | *B. melitensis* | - | 0 | - | 0 | 0 | 0 | 0 | 0 | 0 | 0 | 0 | 0 | 0 | 0 | 0 | 0 | - | 0 | melitensis | --- | AFIP |
| 9916388 | *B. melitensis* | - | 0 | 0 | 0 | 0 | 0 | 0 | 0 | 0 | 0 | 0 | 0 | 0 | 0 | 0 | 0 | 0 | 0 | melitensis | --- | AFIP |
| 9916395* | *B. melitensis* | - | 0 | 0 | 0 | 0 | 0 | 0 | 0 | 0 | 0 | 0 | 0 | 0 | 0 | 0 | 0 | 0 | 0 | melitensis | --- | AFIP |
| 9916395* | *B. melitensis* | - | 0 | - | 0 | 0 | 0 | 0 | 0 | 0 | 0 | 0 | 0 | 0 | 0 | 0 | 0 | - | - | melitensis | --- | AFIP |
| 9916397 | *B. melitensis* | - | 0 | - | 0 | 0 | 0 | 0 | 0 | 0 | 0 | 0 | 0 | 0 | 0 | 0 | 0 | - | - | melitensis | --- | AFIP |
| 9916693* | *B. melitensis* | - | 0 | 0 | 0 | 0 | 0 | 0 | 0 | 0 | 0 | 0 | 0 | 0 | 0 | 0 | 0 | 0 | 0 | melitensis | --- | AFIP |
| 4942* | *B. melitensis* | - | 0 | 0 | 0 | 0 | 0 | 0 | 0 | 0 | 0 | 0 | 0 | 0 | 0 | 0 | 0 | 0 | 0 | melitensis | --- | AFIP |
| Cal/Dolphin 1312* | *B. ceti* | - | 0 | 0 | 0 | 0 | 0 | 0 | 0 | 0 | 0 | 0 | 0 | 0 | 0 | 0 | 0 | 0 | 0 | no derived | CA, USA | AFIP |
| 7-269 1523 | *B. pinnipedialis* | - | 0 | 0 | 0 | 0 | 0 | 0 | 0 | 0 | 0 | 0 | 0 | 0 | 0 | 0 | 0 | 0 | 0 | no derived | --- | AFIP |
| 54/94 1491 | *B. pinnipedialis* | - | 0 | 0 | 0 | 0 | 0 | 0 | 0 | 0 | 0 | 0 | 0 | 0 | 0 | 0 | 0 | 0 | 0 | no derived | UK | AFIP |
| 40/94 1509 | *B. pinnipedialis* | - | 0 | - | 0 | 0 | 0 | 0 | 0 | 0 | 0 | 0 | 0 | 0 | 0 | 0 | 0 | - | - | no derived | UK | AFIP |
| 7-324* | *B. pinnipedialis* | - | 0 | 0 | 0 | 0 | 0 | 0 | 0 | 0 | 0 | 0 | 0 | 0 | 0 | 0 | 0 | 0 | 0 | no derived | --- | AFIP |
| 6-408 1521 | *B. pinnipedialis* | - | 0 | 0 | 0 | 0 | 0 | 0 | 0 | 0 | 0 | 0 | 0 | 0 | 0 | 0 | 0 | - | - | no derived | --- | AFIP |
| Dolphin 1258 | *B. ceti* | - | 0 | 0 | 0 | 0 | 0 | 0 | 0 | 0 | 0 | 0 | 0 | 0 | 0 | 0 | 0 | 0 | 0 | no derived | --- | AFIP |
| Dol/Plac | *B. ceti* | - | 0 | 0 | - | 0 | 0 | 0 | 0 | 0 | 0 | 0 | 0 | 0 | 0 | 0 | 0 | 0 | 0 | no derived | --- | AFIP |
| 44/94* | *B. pinnipedialis* | - | 0 | - | 0 | 0 | 0 | 0 | 0 | 0 | 0 | 0 | 0 | 0 | 0 | 0 | 0 | - | - | no derived | UK | AFIP |
| 48/94 | *B. pinnipedialis* | - | 0 | 0 | 0 | 0 | 0 | 0 | 0 | 0 | 0 | 0 | 0 | 0 | 0 | 0 | 0 | 0 | 0 | no derived | UK | AFIP |
| 6-566 1522 | *B. pinnipedialis* | - | 0 | - | 0 | 0 | 0 | 0 | 0 | 0 | 0 | 0 | 0 | 0 | 0 | 0 | 0 | - | - | no derived | --- | AFIP |
| BD0018 | *B. canis* | - | 0 | 0 | 0 | 0 | 0 | 0 | 0 | 0 | 0 | 0 | 0 | 0 | 0 | 0 | 0 | 1 | 1 | J | --- | BDAP |
| BD0019* | *B. abortus* | - | 0 | - | 1 | 1 | 1 | 0 | 0 | - | 1 | 0 | 0 | 0 | 0 | 0 | 0 | - | 0 | B | --- | BDAP |
| BD0021 | *B. abortus* | - | 0 | 1 | 1 | | 1 | 0 | 0 | 1 | 1 | 0 | 0 | 0 | 0 | - | 0 | - | - | B | --- | BDAP |
| BD0022* | *B. suis* | - | 0 | 0 | - | 0 | 0 | 0 | 0 | 0 | 0 | 0 | 0 | 0 | 0 | 0 | 0 | 0 | 0 | suis | --- | BDAP |
| BD0023 | *B. melitensis* | - | 0 | 0 | 0 | 0 | 0 | 0 | 0 | 0 | 0 | 0 | 0 | 0 | 0 | 0 | - | 0 | 0 | melitensis | --- | BDAP |
| BD0024 | *B. neotomae* | - | 0 | - | 0 | 0 | 0 | 0 | 0 | 0 | 0 | 0 | 0 | 0 | 0 | 0 | 0 | - | 0 | no derived | --- | BDAP |
| BD0025* | *B. suis* | - | 0 | 0 | 0 | 0 | 0 | 0 | 0 | 0 | 0 | 0 | 0 | 0 | 0 | 0 | 0 | 1 | 1 | J | --- | BDAP |
| BD0026 | *B. neotomae* | - | 0 | 0 | 0 | 0 | 0 | 0 | 0 | 0 | 0 | 0 | 0 | 0 | 0 | 0 | 0 | 0 | 0 | no derived | --- | BDAP |
| BD0027* | *B. melitensis* | - | 0 | 0 | 0 | 0 | 0 | 0 | 0 | 0 | 0 | 0 | 0 | 0 | 0 | 0 | 0 | - | 0 | melitensis | --- | BDAP |
| BD0028 | *B. suis* | - | 0 | 0 | 0 | 0 | 0 | 0 | 0 | 0 | 0 | 0 | 0 | 0 | 0 | 1 | 1 | 0 | 0 | I | --- | BDAP |
| B. abortus #1 | *B. abortus* | - | 0 | 0 | 0 | 0 | 0 | 0 | 0 | 0 | 0 | 0 | 0 | 0 | 0 | 0 | 0 | 0 | 0 | abortus | AK, USA | APHL |
| B. abortus #2 | *B. abortus* | - | 0 | 0 | 0 | - | 1 | 0 | 0 | 1 | 1 | 0 | 0 | 0 | 0 | 0 | 0 | 0 | 0 | C | AK, USA | APHL |
| B. ANMC #3 | *B. canis* | - | 0 | 0 | 0 | 0 | 0 | 0 | 0 | 0 | 0 | 0 | 0 | 0 | 0 | 0 | 0 | 1 | 1 | J | AK, USA | APHL |
| B. ANMC37 #4 | *B. canis* | - | 0 | 0 | 0 | 0 | 0 | 0 | 0 | 0 | 0 | 0 | 0 | 0 | 0 | 0 | 0 | 1 | 1 | J | AK, USA | APHL |
| B. canis #5 | *B. canis* | - | 0 | 0 | - | 0 | 0 | 0 | 0 | 0 | 0 | 0 | 0 | 0 | 0 | - | 0 | 1 | 1 | J | AK, USA | APHL |
| B. melitensis #6 | *B. melitensis* | - | 0 | 0 | 0 | 0 | 0 | 0 | 0 | 0 | 0 | 0 | 0 | 0 | 0 | 0 | 0 | 0 | 0 | melitensis | AK, USA | APHL |
| B. suis #7* | *B. suis* | - | 0 | 0 | - | 0 | 0 | 0 | 0 | 0 | 0 | 0 | 0 | 0 | 0 | - | 0 | 0 | 0 | suis | AK, USA | APHL |
| BA 7 | *B. abortus* | - | 0 | 0 | 0 | 0 | 0 | - | 1 | 1 | 1 | - | 0 | 0 | 0 | - | 0 | 0 | 0 | D | --- | unknown |
| Canis5* | *B. canis* | - | 0 | 0 | - | 0 | - | - | - | 0 | - | - | - | - | - | - | - | 1 | 1 | J | --- | unknown |
| 67080 | *B. melitensis* | - | 0 | 0 | 0 | 0 | 0 | 0 | 0 | 0 | 0 | 0 | - | 1 | 1 | - | 0 | 0 | 0 | G | --- | unknown |
| E8117 | *B. melitensis* | 2 | 0 | 0 | 0 | 0 | 0 | 0 | 0 | 0 | 0 | 0 | 0 | 0 | 1 | 0 | 0 | 0 | 0 | H | IL, USA | unknown |
| G8551 | *B. melitensis* | - | 0 | 0 | 0 | 0 | 0 | 0 | 0 | 0 | 0 | 0 | 0 | 0 | 1 | 0 | 0 | 0 | 0 | H | IL, USA | unknown |
| F0531 | *B. suis* | 1 | 0 | 0 | - | 0 | 0 | 0 | 0 | 0 | 0 | 0 | 0 | 0 | 0 | 1 | - | 0 | 0 | I | IL, USA | unknown |
| 2308MR | *B. abortus* | 1 | 0 | 1 | 1 | - | 1 | 0 | 0 | 1 | 1 | 0 | 0 | 0 | 0 | 0 | 0 | 0 | 0 | B | --- | unknown |
| Neotomae7 | *B. neotomae* | - | 0 | 0 | 0 | 0 | 0 | 0 | 0 | 0 | 0 | 0 | 0 | 0 | 0 | 0 | 0 | 0 | 0 | no derived | --- | unknown |
| Bm6 | *B. melitensis* | - | 0 | 0 | 0 | 0 | 0 | 0 | 0 | 0 | 0 | 0 | 0 | 0 | 0 | 0 | 0 | 0 | 0 | melitensis | --- | unknown |
| REV1 | *B. melitensis* | - | 0 | 0 | 0 | 0 | 0 | 0 | 0 | 0 | 0 | 0 | 0 | 0 | 1 | 0 | 0 | 0 | 0 | H | --- | unknown |
| PHE1 | *B. abortus* | 1 | 0 | 1 | 1 | - | 1 | 0 | 0 | 1 | 1 | - | 0 | 0 | 0 | 0 | 0 | 0 | 0 | B | --- | unknown |
| Abortus2308 | *B. abortus* | 1 | 0 | 1 | 1 | - | 1 | 0 | 0 | 1 | 1 | 0 | 0 | 0 | 0 | 0 | 0 | 0 | 0 | B | --- | unknown |
| B.suis23444 | *B. suis* | 1 | 0 | 0 | 0 | 0 | 0 | 0 | 0 | 0 | 0 | 0 | 0 | 0 | 0 | 1 | 1 | 0 | 0 | I | --- | Battelle |
| RB51 | *B. abortus* | 1 | 0 | - | 0 | - | 1 | 0 | 0 | - | 1 | 0 | 0 | 0 | 0 | 0 | 0 | - | - | C | --- | BYU |
| 2016 #1001* | *B. abortus* | 1 | 0 | 0 | 0 | 0 | 0 | - | 1 | 1 | 1 | 0 | 0 | 0 | 0 | 0 | 0 | 0 | 0 | D | MS, USA | BYU |
| 2018 #1002 | *B. abortus* | 1 | 0 | 0 | 0 | 0 | 0 | - | 1 | 1 | 1 | 0 | 0 | 0 | 0 | 0 | 0 | 0 | 0 | D | CA, USA | BYU |
| 2019 #1003 | *B. abortus* | 1 | 0 | 0 | 0 | 0 | 0 | - | 1 | 1 | 1 | 0 | 0 | 0 | 0 | 0 | 0 | 0 | 0 | D | CA, USA | BYU |
| 2033 #1005 | *B. abortus* | 1 | 0 | 0 | 0 | 0 | 0 | - | 1 | 1 | 1 | 0 | 0 | 0 | 0 | 0 | 0 | 0 | 0 | D | MO, USA | BYU |
| 2042 #1006* | *B. abortus* | 1 | 0 | 1 | 1 | 1 | 1 | 0 | 0 | 1 | 1 | - | 0 | 0 | 0 | 0 | 0 | | 0 | B | FL, USA | BYU |
| 2045 #1007* | *B. abortus* | 1 | 0 | 0 | 0 | 0 | 0 | 1 | 1 | - | 1 | 0 | 0 | 0 | 0 | 0 | 0 | 0 | 0 | D | FL, USA | BYU |

| | | | | | | | | | | | | | | | | | | | | | | |
|---|---|---|---|---|---|---|---|---|---|---|---|---|---|---|---|---|---|---|---|---|---|---|
| 2051 #1011* | *B. abortus* | 1 | 0 | 1 | 1 | - | 1 | 0 | 0 | 1 | 1 | 0 | 0 | 0 | 0 | 0 | - | - | 0 | B | OK, USA | BYU |
| 1135 #aej* | *B. abortus* | - | 0 | 1 | 1 | - | 1 | 0 | 0 | 1 | 1 | 0 | 0 | 0 | 0 | 0 | 0 | 0 | 0 | B | --- | BYU |
| 2308 #772 | *B. abortus* | 1 | 0 | 1 | 1 | 1 | 1 | 0 | 0 | 1 | 1 | 0 | 0 | 0 | 0 | 0 | 0 | 0 | 0 | B | --- | BYU |
| 00-666 #1668* | *B. abortus* | 4 | 0 | 0 | 0 | 1 | 1 | 0 | 0 | 1 | 1 | 0 | 0 | 0 | 0 | 0 | 0 | 0 | 0 | C | WY, USA | BYU |
| 00-671 #1669* | *B. abortus* | 4 | 0 | 0 | 0 | 1 | 1 | 0 | 0 | 1 | 1 | 0 | 0 | 0 | 0 | 0 | 0 | 0 | 0 | C | WY, USA | BYU |
| 0-508 #1218* | *B. abortus* | 1 | 0 | 0 | 0 | 0 | 0 | - | 1 | 1 | 1 | 0 | 0 | 0 | 0 | 0 | 0 | 0 | 0 | D | ND, USA | BYU |
| 0-642 #1217* | *B. abortus* | 1 | 0 | 0 | 0 | 0 | 0 | 1 | 1 | 1 | 1 | 0 | 0 | 0 | 0 | 0 | 0 | 0 | 0 | D | ND, USA | BYU |
| 19 #1135* | *B. abortus* | 1 | 0 | 1 | 1 | 1 | 1 | 0 | 0 | 1 | 1 | 0 | 0 | 0 | 0 | 0 | 0 | - | - | B | --- | BYU |
| 2040 #1020* | *B. abortus* | 1 | 0 | 0 | 0 | 0 | 0 | 1 | 1 | 1 | 1 | 0 | 0 | 0 | 0 | 0 | 0 | 0 | 0 | D | TN, USA | BYU |
| 2052 #1012* | *B. abortus* | 1 | 0 | 1 | 1 | 1 | 1 | 0 | 0 | 1 | 1 | 0 | 0 | 0 | 0 | 0 | 0 | 0 | 0 | B | OK, USA | BYU |
| 2054 #1021* | *B. abortus* | 1 | 0 | 1 | 1 | 1 | - | 0 | 0 | 1 | 1 | 0 | 0 | 0 | 0 | 0 | 0 | 0 | 0 | B | NM, USA | BYU |
| 2073 #1022* | *B. abortus* | 1 | 0 | 1 | 1 | 1 | 1 | 0 | 0 | 1 | 1 | 0 | 0 | 0 | 0 | 0 | 0 | 0 | 0 | B | GA, USA | BYU |
| 2100 #1026* | *B. abortus* | 1 | 0 | 0 | 0 | 0 | 0 | 1 | 1 | 1 | 1 | 0 | 0 | 0 | 0 | 0 | 0 | 0 | 0 | D | GA, USA | BYU |
| 2-1230 #1206 | *B. abortus* | 1 | 0 | 0 | 0 | 0 | 0 | 1 | 1 | 1 | 1 | 0 | 0 | 0 | 0 | 0 | 0 | 0 | 0 | D | MT, USA | BYU |
| 2158 #1048* | *B. abortus* | 1 | 0 | 0 | 0 | 1 | 1 | 0 | 0 | 1 | 1 | 0 | 0 | 0 | 0 | 0 | 0 | 0 | 0 | C | KS, USA | BYU |
| 2256 #1042* | *B. abortus* | 1 | 0 | 0 | 0 | 0 | 0 | 1 | 1 | 1 | 1 | 0 | 0 | 0 | 0 | 0 | 0 | 0 | 0 | D | AZ, USA | BYU |
| 2257 #1043* | *B. abortus* | 1 | 0 | 0 | 0 | 0 | 0 | 1 | 1 | 1 | 1 | 0 | 0 | 0 | 0 | 0 | 0 | 0 | 0 | D | AR, USA | BYU |
| 2471 #1090* | *B. abortus* | 1 | 0 | 0 | 0 | 0 | 0 | 1 | 1 | 1 | 1 | 0 | 0 | 0 | 0 | 0 | 0 | 0 | 0 | D | KY, USA | BYU |
| 3 #1332* | *B. abortus* | 1 | 0 | 0 | 0 | 0 | 0 | 1 | 1 | 1 | 1 | 0 | 0 | 0 | 0 | 0 | 0 | 0 | 0 | D | KS, USA | BYU |
| 544 #698 | *B. abortus* | 1 | 0 | 0 | 0 | 1 | 1 | 0 | 0 | 1 | 1 | 0 | 0 | 0 | 0 | 0 | 0 | 0 | 0 | C | --- | BYU |
| 8-953 #1146 | *B. abortus* | 1 | 0 | 0 | 0 | 0 | 0 | 1 | 1 | 1 | 1 | 0 | 0 | 0 | 0 | 0 | 0 | 0 | 0 | D | MT, USA | BYU |
| 1-107 #1107* | *B. canis* | - | 0 | 0 | - | 0 | 0 | 0 | 0 | 0 | 0 | 0 | 0 | 0 | 0 | 0 | 0 | 1 | 1 | J | MO, USA | BYU |
| 1-271 #1115* | *B. canis* | - | 0 | 0 | 0 | 0 | 0 | 0 | 0 | 0 | 0 | 0 | 0 | 0 | 0 | 0 | 0 | 1 | 1 | J | MO, USA | BYU |
| 1-324 #1109* | *B. canis* | - | 0 | 0 | 0 | 0 | 0 | 0 | 0 | 0 | 0 | 0 | 0 | 0 | 0 | 0 | 0 | 1 | 1 | J | MO, USA | BYU |
| 5K33 #1107* | *B. neotomae* | - | 0 | 0 | 0 | 0 | 0 | 0 | 0 | 0 | 0 | 0 | 0 | 0 | 0 | 0 | 0 | 0 | 0 | no derived | USA | BYU |
| 1-139 #1113* | *B. ovis* | - | 0 | 0 | 0 | 0 | 0 | 0 | 0 | 0 | 0 | 0 | 0 | 0 | 0 | 0 | 0 | 0 | 0 | no derived | KS, USA | BYU |
| 1-507 #1117 | *B. ovis* | - | 0 | 0 | 0 | 0 | 0 | 0 | 0 | 0 | 0 | 0 | 0 | 0 | 0 | 0 | 0 | 0 | 0 | no derived | GA, USA | BYU |
| 63/290 #1155 | *B. ovis* | - | 0 | 0 | 0 | 0 | 0 | 0 | 0 | 0 | 0 | 0 | 0 | 0 | 0 | 0 | 0 | 0 | 0 | no derived | USA | BYU |
| 40/67 #1154 | *B. suis* | 4 | 0 | 0 | - | 0 | 0 | 0 | 0 | 0 | 0 | 0 | 0 | 0 | 0 | 0 | 0 | 1 | 1 | J | USA | BYU |
| 1-138 #1108* | *B. suis* | 1 | 0 | 0 | - | 0 | 0 | 0 | 0 | 0 | 0 | 0 | 0 | 0 | 0 | - | 1 | 0 | 0 | I | NJ, USA | BYU |
| 2366 #1071* | *B. suis* | 1 | 0 | 0 | - | 0 | 0 | 0 | 0 | 0 | 0 | 0 | 0 | 0 | 0 | - | 1 | 0 | 0 | I | IA, USA | BYU |
| 2367 #1072 | *B. suis* | 1 | 0 | 0 | - | 0 | 0 | 0 | 0 | 0 | 0 | 0 | 0 | 0 | 0 | - | 1 | 0 | 0 | I | IA, USA | BYU |
| 2483 #1103 | *B. suis* | 1 | 0 | 0 | 0 | 0 | 0 | 0 | 0 | 0 | 0 | 0 | 0 | 0 | 0 | - | 1 | 0 | 0 | I | SC, USA | BYU |
| 40 #1154(2) | *B. suis* | 4 | 0 | 0 | - | 0 | 0 | 0 | 0 | 0 | 0 | 0 | 0 | 0 | 0 | 0 | 0 | 0 | 0 | suis | USA | BYU |
| ATCC23444* | *B. suis* | 1 | 0 | 0 | - | 0 | 0 | 0 | 0 | 0 | 0 | 0 | 0 | 0 | 0 | - | 1 | 0 | 0 | I | --- | BYU |
| ATCC23447 | *B. suis* | 4 | 0 | 0 | 0 | 0 | 0 | 0 | 0 | 0 | 0 | 0 | 0 | 0 | 0 | 0 | 0 | 1 | 1 | J | --- | BYU |
| Thomsen #1152 | *B. suis* | 2 | 0 | 0 | 0 | 0 | 0 | 0 | 0 | 0 | 0 | 0 | 0 | 0 | 0 | 0 | 0 | 0 | 0 | suis | USA | BYU |
| BA1 | *B. abortus* | - | 0 | 0 | 0 | 0 | 0 | 0 | 0 | 0 | 0 | 0 | 0 | 0 | 0 | 0 | 0 | 0 | 0 | abortus | AZ, USA | ADHS |
| BA2 | *B. abortus* | - | 0 | 0 | 0 | 0 | 0 | 0 | 0 | 1 | | - | 0 | 0 | 0 | - | - | - | 0 | E | AZ, USA | ADHS |
| BM1 | *B. melitensis* | - | 0 | 0 | 0 | 0 | 0 | 0 | 0 | 0 | 0 | 0 | 0 | 0 | 0 | 0 | - | 0 | 0 | melitensis | AZ, USA | ADHS |
| BM2 | *B. melitensis* | - | 0 | 0 | 0 | 0 | 0 | 0 | 0 | 0 | 0 | 0 | 0 | 0 | 0 | 0 | 0 | 0 | 0 | melitensis | AZ, USA | ADHS |
| BM3 | *B. melitensis* | - | 0 | 0 | 0 | 0 | 0 | 0 | 0 | 0 | 0 | 0 | 0 | 0 | 1 | 0 | 0 | 0 | 0 | H | AZ, USA | ADHS |
| BM4 | *B. melitensis* | - | 0 | 0 | 0 | 0 | 0 | 0 | 0 | 0 | 0 | 0 | 1 | 1 | 1 | 0 | 0 | 0 | 0 | G | AZ, USA | ADHS |
| BS1 | *B. suis* | - | 0 | 0 | - | 0 | 0 | 0 | 0 | 0 | 0 | 0 | 0 | 0 | 0 | - | 0 | 0 | 0 | suis | AZ, USA | ADHS |
| 2007008533 | *B. abortus* | 1 | 0 | 0 | 0 | 0 | 0 | 0 | 0 | 1 | 1 | 0 | 0 | 0 | 0 | 0 | 0 | 0 | 0 | E | USA | CDC |
| 2006001739 | *B. abortus* | 1 | 0 | 1 | 1 | - | 1 | 0 | 0 | 1 | 1 | 0 | 0 | 0 | 0 | - | - | 0 | 0 | B | USA | CDC |
| 2002034574 | *B. abortus* | 4 | 0 | 1 | | - | 1 | 0 | 0 | 1 | | 0 | 0 | 0 | 0 | 0 | 0 | - | 0 | B | USA | CDC |
| 2002026227 | *B. abortus* | 5 | 0 | 0 | 0 | 0 | 0 | 0 | 0 | 0 | 0 | 0 | 0 | 0 | 0 | 0 | 0 | 0 | 0 | abortus | USA | CDC |
| 2002034902 | *B. abortus* | 1 | 0 | 1 | - | - | 1 | 0 | 0 | 1 | 1 | 0 | 0 | 0 | 0 | 0 | 0 | 0 | 0 | B | USA | CDC |
| 2002034960 | *B. abortus* | 1 | 0 | 1 | - | - | - | 0 | 0 | 1 | 1 | 0 | 0 | 0 | 0 | 0 | 0 | 0 | 0 | B | USA | CDC |
| 2004017504 | *B. abortus* | 2 | 0 | 0 | 0 | - | 1 | 0 | 0 | 1 | 1 | 0 | 0 | 0 | 0 | 0 | 0 | 0 | 0 | C | USA | CDC |
| 2006014040 | *B. abortus* | 1 | 0 | 1 | 1 | 1 | 1 | 0 | 0 | 1 | 1 | 0 | 0 | 0 | 0 | 0 | 0 | 0 | 0 | B | USA | CDC |
| 2002000613 | *B. melitensis* | 1 | 0 | 0 | 0 | 0 | 0 | 0 | 0 | 0 | 0 | 0 | 0 | 0 | 0 | 0 | - | 0 | 0 | melitensis | USA | CDC |
| 2001038790 | *B. melitensis* | 3 | 0 | 0 | 0 | 0 | 0 | 0 | 0 | 0 | 0 | 0 | 0 | 0 | 0 | 0 | - | 0 | 0 | melitensis | USA | CDC |
| 2001034489 | *B. melitensis* | 2 | 0 | 0 | 0 | 0 | 0 | 0 | 0 | 0 | 0 | 0 | 0 | 0 | 1 | 0 | 0 | 0 | 0 | H | USA | CDC |
| 2000031719 | *B. melitensis* | 2 | - | 0 | 0 | 0 | 0 | 0 | 0 | - | 0 | 0 | 0 | - | 1 | 0 | 0 | 0 | 0 | H | USA | CDC |
| 2002020524 | *B. melitensis* | 3 | 0 | 0 | 0 | 0 | 0 | 0 | 0 | 0 | 0 | 0 | 0 | 0 | 0 | 0 | - | 0 | 0 | melitensis | USA | CDC |
| 2004017502 | *B. melitensis* | 1 | 0 | 0 | 0 | 0 | 0 | 0 | 0 | 0 | 0 | 0 | 0 | 0 | 0 | 0 | - | 0 | 0 | melitensis | USA | CDC |
| 2006004348 | *B. ovis* | - | 0 | 0 | 0 | 0 | 0 | 0 | 0 | 0 | 0 | 0 | 0 | 0 | 0 | 0 | 0 | 0 | 0 | no derived | USA | CDC |
| 2002013049 | *B. ovis* | - | 0 | 0 | 0 | 0 | - | 0 | 0 | 0 | 0 | 0 | 0 | 0 | 0 | 0 | 0 | 0 | 0 | no derived | USA | CDC |
| 2003007064 | *B. ovis* | - | 0 | 0 | 0 | 0 | - | - | - | 0 | - | 0 | 0 | 0 | 0 | 0 | 0 | 0 | 0 | no derived | USA | CDC |
| 2004017518 | *B. ovis* | - | 0 | 0 | 0 | 0 | 0 | 0 | 0 | 0 | 0 | 0 | 0 | 0 | 0 | 0 | 0 | 0 | 0 | no derived | USA | CDC |
| 2007005564 | *B. suis* | 1 | 0 | 0 | - | 0 | 0 | 0 | 0 | 0 | 0 | 0 | 0 | 0 | 0 | 1 | - | 0 | 0 | I | USA | CDC |
| 2005013583 | *B. suis* | 1 | 0 | 0 | 0 | 0 | 0 | 0 | 0 | 0 | 0 | 0 | 0 | 0 | 0 | 1 | 1 | 0 | 0 | I | USA | CDC |
| 2005013756 | *B. suis* | 1 | 0 | 0 | - | 0 | 0 | 0 | 0 | 0 | 0 | 0 | 0 | 0 | 0 | 1 | - | 0 | 0 | I | USA | CDC |
| 2004017627 | *B. suis* | 1 | 0 | 0 | - | 0 | 0 | 0 | 0 | 0 | 0 | 0 | 0 | 0 | 0 | - | - | 0 | 0 | no call | USA | CDC |
| #0122036 | *B. melitensis* | - | 0 | 0 | 0 | 0 | 0 | 0 | 0 | 0 | 0 | 0 | 0 | 0 | 1 | - | - | 0 | 0 | H | AZ, USA | ADHS |
| #01043503 | *B. melitensis* | - | 0 | 0 | 0 | 0 | 0 | 0 | 0 | 0 | 0 | 0 | 0 | 0 | 1 | 0 | 0 | 0 | 0 | H | AZ, USA | ADHS |
| ID04-0970002449 | *B. melitensis* | - | 0 | 0 | 0 | 0 | - | - | - | 0 | - | 0 | - | 0 | - | - | - | - | 0 | no call | CA, USA | ADHS |
| ID05-2010006921 | *B. melitensis* | - | 0 | 0 | 0 | 0 | 0 | 0 | 0 | 0 | 0 | 0 | 0 | 0 | 1 | - | 0 | 0 | 0 | H | AZ, USA | ADHS |
| BT06-1520000049 | *B. suis* | - | 0 | 0 | - | 0 | 0 | 0 | 0 | 0 | 0 | 0 | 0 | 0 | 0 | - | - | 0 | 0 | no call | AZ, USA | ADHS |

| | | | | | | | | | | | | | | | | | | | | | | |
|---|---|---|---|---|---|---|---|---|---|---|---|---|---|---|---|---|---|---|---|---|---|---|
| BT06-2340000116 | *B. melitensis* | - | 0 | 0 | 0 | 0 | 0 | 0 | 0 | 0 | 0 | 0 | 0 | 0 | 1 | - | - | - | 0 | H | AZ, USA | ADHS |
| BT07-0360000003 | *B. melitensis* | - | - | 0 | - | 0 | 0 | 0 | 0 | - | 0 | - | 0 | - | 0 | - | - | - | 0 | melitensis | AZ, USA | ADHS |
| BT07-0640000010 | *B. melitensis* | - | 0 | 0 | 0 | 0 | 0 | 0 | 0 | 0 | 0 | - | 1 | 1 | 1 | 0 | 0 | 0 | 0 | G | AZ, USA | ADHS |
| PB07-115001 | *B. abortus* | - | 0 | 1 | | - | 1 | 0 | 0 | 1 | 1 | - | 0 | 0 | 0 | 0 | 0 | 0 | 0 | B | AZ, USA | ADHS |
| PB07-221001 | *B. melitensis* | - | 0 | 0 | - | - | 0 | - | 0 | - | - | - | - | - | 1 | - | - | - | 0 | H | AZ, USA | ADHS |
| ID05 | *B. canis* | - | 0 | 0 | - | 0 | 0 | 0 | 0 | 0 | 0 | 0 | 0 | 0 | 0 | 0 | 0 | 1 | 1 | J | AZ, USA | ADHS |
