# Supplementary material for: Genotyping of Brucella species using clade specific SNPs
Source: BMC Microbiol. 2012 Jun 19;12:110. doi: 10.1186/1471-2180-12-110 (PMC3747857; doi:10.1186/1471-2180-12-110)
Supplement: Additional file 3 Table S1. — List of 28 whole genomes used forin silicocomparisons to SNP alleles from MIP assay. (PDF 62 kb). [file 1471-2180-12-110-S3.docx]

|  | | | |
| --- | --- | --- | --- |
| **Additional file 3: Table S1** List of 28 whole genomes used for *in silico* comparisons to SNP alleles from MIP assay. | | | |
|  |  |  |  |
| **Species** | **Strain** | **Biovar** | **GenBank Accessions** |
| *Brucella abortus* | 2308 | 1 | AM040264, AM040265 |
| *Brucella abortus* | 9-941 | 1 | AE017223, AE017224 |
| *Brucella abortus* | S19 | 1 | CP000887, CP000888 |
| *Brucella abortus* | 86/8/59 | 2 | ACBJ00000000 |
| *Brucella abortus* | Tulya | 3 | ACBI00000000 |
| *Brucella abortus* | 292 | 4 | ACBH00000000 |
| *Brucella abortus* | 870 | 6 | ACBG00000000 |
| *Brucella abortus* | C68 | 9 | ACEL00000000 |
| *Brucella canis* | RM6/66 |  | CP000872, CP000873 |
| *Brucella ceti* | B1/94 |  | ACEK00000000 |
| *Brucella ceti* | M13/05/1 |  | ACBP00000000 |
| *Brucella ceti* | M490/95/1 |  | ACEJ00000000 |
| *Brucella ceti* | M644/93/1 |  | ACBO00000000 |
| *Brucella ceti* | F5/99 |  | ACFF00000000 |
| *Brucella melitensis* | 16M | 1 | AE008917, AE008918 |
| *Brucella melitensis* | Rev. 1 | 1 | ACEG00000000 |
| *Brucella melitensis* | 63/9 | 2 | CP001488, CP001489 |
| *Brucella melitensis* | Ether | 3 | ACEI00000000 |
| *Brucella neotomae* | 5K33 |  | ACEH00000000 |
| *Brucella ovis* | 63/290, ATCC 25840 |  | CP000709, CP000708 |
| *Brucella pinnipedialis* | B2/94 |  | ACBN00000000 |
| *Brucella pinnipedialis* | M292/94/1 |  | ACEF00000000 |
| *Brucella* sp. | 83/13 |  | ACBQ00000000 |
| *Brucella suis* | 1330 | 1 | AE014291, AE014292 |
| *Brucella suis* | Thomsen, ATCC 23445 | 2 | CP000911, CP000912 |
| *Brucella suis* | 686 | 3 | ACBL00000000 |
| *Brucella suis* | 40 | 4 | ACJK00000000 |
| *Brucella suis* | 513 | 5 | ACBK00000000 |
