# Supplementary material for: Genotyping of Brucella species using clade specific SNPs
Source: BMC Microbiol. 2012 Jun 19;12:110. doi: 10.1186/1471-2180-12-110 (PMC3747857; doi:10.1186/1471-2180-12-110)
Supplement: Additional file 4 Table S2. — List ofBrucellaisolates used in 17 CUMA assays, including isolate name, species, and biovar when known or applicable and the SNP allele for each assay. (PDF 44 kb). [file 1471-2180-12-110-S4.docx]

| **Additional file 4: Table S2** List of *Brucella* isolates used in 17 CUMA assays, listed by biovar when known or applicable. | | | | | | | | | | |
| --- | --- | --- | --- | --- | --- | --- | --- | --- | --- | --- |
|  |  |  |  |  |  |  |  |  |  |  |
|  | Biovar | | | | | | | | |  |
| Species | 1 | 2 | 3 | 4 | 5 | 6 | 7 | 9 | Unknown or N/A | Total |
| *B. abortus* | 39 | 2 | 1 | 4 | 2 | 1 | 1 | 1 | 23 | 74 |
| *B. canis* |  |  |  |  |  |  |  |  | 13 | 13 |
| *B. melitensis* | 52 | 16 | 23 |  |  |  |  |  | 85 | 176 |
| *B. neotomae* |  |  |  |  |  |  |  |  | 6 | 6 |
| *B. ovis* |  |  |  |  |  |  |  |  | 8 | 8 |
| *B. suis* | 28 | 1 |  | 4 |  |  |  |  | 19 | 52 |
| *B. ceti* |  |  |  |  |  |  |  |  | 3 | 3 |
| *B. pinnipedialis* |  |  |  |  |  |  |  |  | 8 | 8 |
|  |  |  |  |  |  |  |  |  |  | 340 |
